# Steric stabilization of colloidal UiO-66 nanocrystals with oleylammonium octadecylphosphonate†

**DOI:** 10.1039/d4sc06528j

**Published:** 2024-12-02

**Authors:** Sungho V. Park, Lakshmi Bhai, Gahyun Annie Lee, Ah-Hyung Alissa Park, Lauren E. Marbella, Jonathan S. Owen

**Affiliations:** a Department of Chemistry, Columbia University New York NY 10027 USA jso2115@columbia.edu; b Department of Chemical Engineering, Columbia University New York New York 10027 USA; c Lenfest Center for Sustainable Energy, Columbia University New York NY 10027 USA; d Department of Earth and Environmental Engineering, Columbia University New York New York 10027 USA

## Abstract

We report the synthesis and characterization of octahedral UiO-66 nanocrystals (*d* = 17–25 nm) terminated with amine, oleate, and octadecylphosphonate ligands. Acetate capped UiO-66 nanocrystals were dispersed in toluene using oleic acid and oleylamine. Ligand exchange with octadecylphosphonic acid produces ammonium octadecylphosphonate terminated nanocrystals with coverages of 2.6–3.2 chains per nm^2^ that stabilize colloidal dispersions in nonpolar solvents. Liquid phase ^1^H and ^31^P nuclear magnetic resonance (NMR) spectra of the linkers and surface ligands display line shapes that are broadened by slow tumbling of the nanocrystals. Octadecylphosphonate functionalized MOFs have up to ∼30% carbon dioxide absorption capacities compared to bulk UiO-66 after correcting for the ligand mass. These results illustrate the intriguing perspective that MOF nanocrystals can be characterized and manipulated like a macromolecular complex and represent an important milestone in the nascent field of MOF surface science.

## Abstract 2

Surface ligand exchange of toluene-dispersible UiO-66 nanocrystals suggests that these particles can be characterized and manipulated like a macromolecular complex.

## Graphical abstract

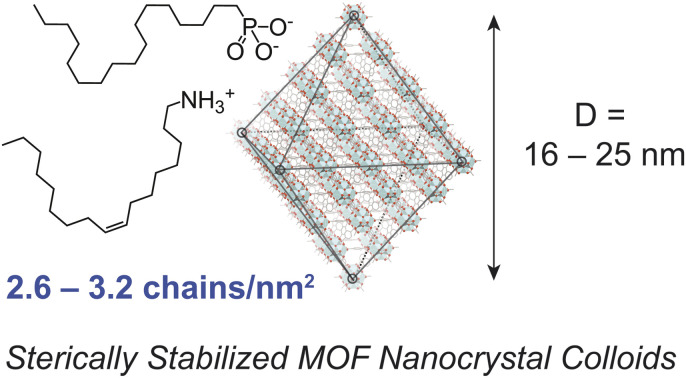

## Introduction

Nanometer scale metal–organic framework crystals (MOF nanocrystals) are desirable for a wide variety of applications in catalysis, separations,^1,2^ and medicine.^3–7^ The high exterior surface area of the nanocrystal increases their catalytic activity^8,9^ and can be used to graft polymers for the synthesis of composite materials.^10,11^ A small crystal size also increases the bioavailability of these materials and their cargo. For these reasons a growing number of publications describe the synthesis and surface modification of MOF nanocrystals, albeit only in polar solvents such as dimethylformamide (DMF) where electrostatically stabilized colloidal dispersions form.^12^

**Fig. 1:**
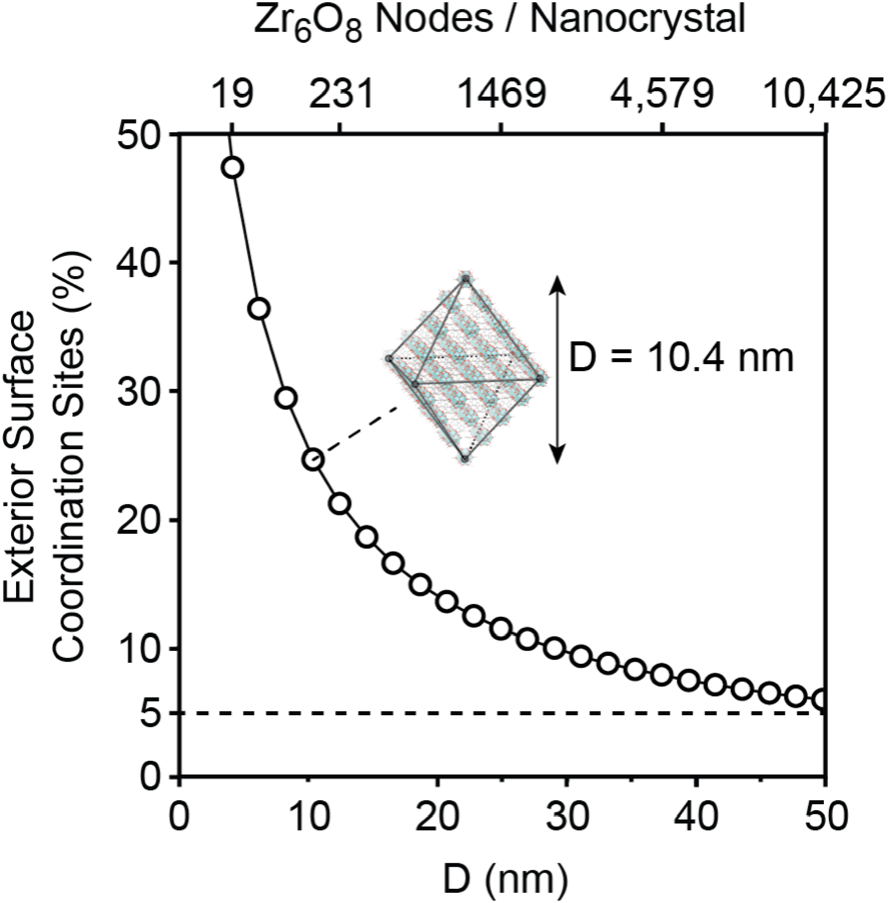
Plot illustrating the increase in the percentage of exterior surface coordination sites as the MOF size decreases. Inset shows a depiction of an example nanocrystal with five nodes along each edge, with body diagonal *D* = 10.4 nm.

As the nanocrystal size shrinks, an increasingly large percentage of the nodal coordination sites are displayed at the nanocrystal's surface. In the case of UiO-66, >5% of all carboxylates in the crystal are found on the exterior surface of nanocrystals less than 50 nm (Fig. 1). The high proportion of surface sites at this length scale facilitates the study of MOF surface coordination chemistry and can improve the performance of polymer-MOF composites, catalysts, and medical technologies.

Syntheses of several nanocrystalline MOFs have been reported, including UiO-66.^13–17^ The nanometer scale size is achieved by introducing high concentrations of monotopic carboxylates, so called “modulators”,^18–21^ and increasing the proportion of linkers beyond the stoichiometric ratio. These conditions produce crystals with zeta potentials sufficiently large to support electrostatically stabilized colloidal dispersions in polar solvents such as methanol and dimethylformamide (DMF) (up to 40 mV).^22,23^ However, under optimized conditions MOF nanocrystals precipitate from the synthesis mixture and are isolated as solids that can be temporarily dispersed by sonication. The dispersibility can be enhanced by choosing solvents that have favorable interactions with the linkers,^22^ and upon introduction of ligands to the nanocrystal's surface. A recent progress report reviews surface modification strategies that address MOF nanoparticle linkers and nodes.^12^ While linker functionalization is more common, the functionalization of nodal sites at framework surfaces is a relatively new endeavor.^24^

Long chain surfactant ligands, calixarenes, and polymers have all been attached to the surface of MOFs post synthesis.^4,25–30^ In some cases, colloidal dispersions in nonpolar solvents (*e.g.*, toluene or chloroform) have been reported with short term stability, despite the relatively large particle sizes typically studied (>100 nm).^26,30^ A recent example functionalized the surfaces of UiO-66 nanocrystals (100–200 nm edge length) with crown ether containing ligands, which provided electrostatically stabilized dispersions in both polar and nonpolar media.^30^ Pure steric stabilization of MOF nanocrystal dispersions, on the other hand, is less common. Phospholipid capped UiO-66 of 25–220 nm has been dispersed in CHCl_3_ and the surface ligand coverage estimated to be 1.3 nm^−2^, which corresponds to approximately 70–80% of the theoretical coverage (1.86 ligands per nm^2^ on the [100] surface or 1.61 ligands per nm^2^ on the [111] surface).^25^ However, researchers have struggled to obtain optically clear dispersions with long term stability, possibly due to the fact that these coverages are less than half of typical ligand densities found on colloidal quantum dots or self-assembled monolayers on metal surfaces.^31,32^

Using oleylamine, oleate, and octadecylphosphonate ligands, we targeted sterically stabilized dispersions of MOF nanocrystals with dimensions less than 50 nm. Dispersibility in nonpolar media could lead to highly active catalysts with a homogeneous structure in solution.^33^ The large proportion of surface coordination sites of crystals on this length scale greatly facilitates the study of their surface coordination chemistry, using bulk characterization techniques. We use liquid phase and solid-state nuclear magnetic resonance (NMR) spectroscopy to explore their chemical formulas and monitor the exchange of their surface ligands. We also describe their gas uptake capacity, which is sensitive to the surface coordination chemistry.

## Results and discussion

### Synthesis and functionalization of UiO-66 nanocrystals

**Scheme 1:**
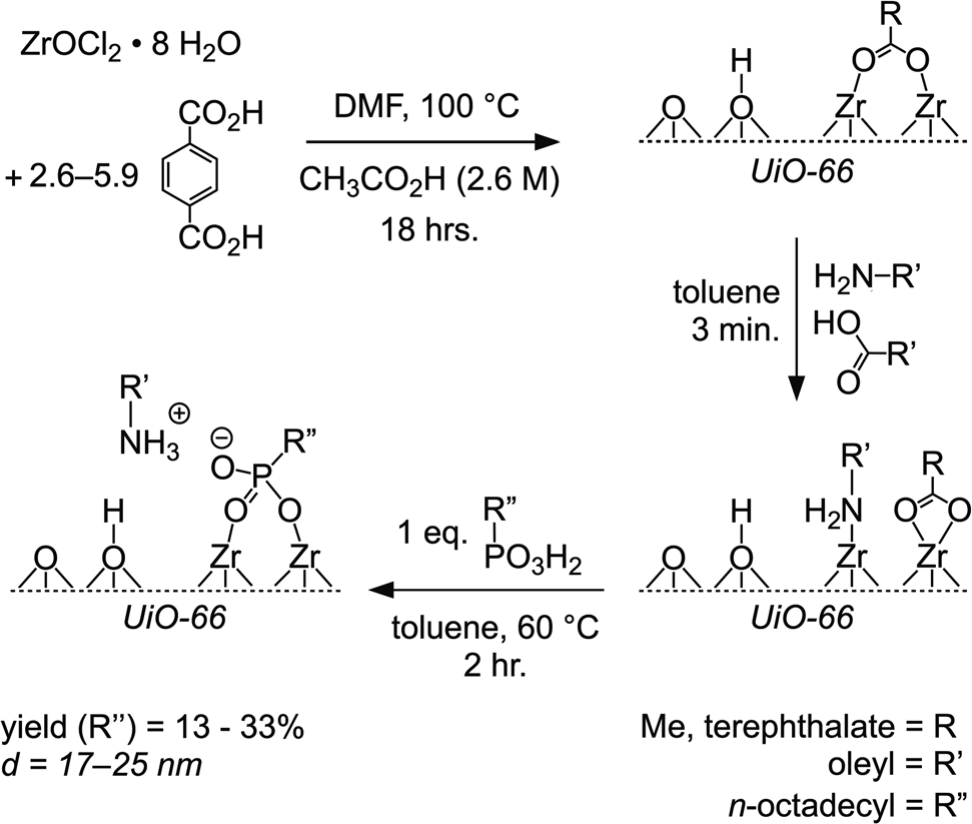
Synthesis and functionalization of UiO-66 nanocrystals illustrating the proposed stoichiometry of the crystal surface during ligand exchange.

UiO-66 nanocrystals were prepared from ZrOCl_2_ as depicted in Scheme 1, following a previous report.^23^ Acetic acid was selected as the modulator as it produces UiO-66 with lower defect densities than other short chain carboxylates^34,35^ Sonication of the terephthalic acid precursor to ensure complete dissolution in DMF was necessary to obtain well faceted and well divided nanocrystals (see ESI†).

The nanocrystal size could be reduced below 100 nm by increasing the concentration of the linker (terephthalic acid), a strategy that was successful in a synthesis of NU-1000 MOF crystals ranging from 300 nm to ∼10 μm in length.^36^ In our hands, the nanocrystal size proved insensitive to the concentration of acetic acid, which appears contrary to previous reports. Instead, if the reaction is conducted without stirring, the acetic acid modulator does influence the nanocrystal size.^23^ The difference between stirred and unstirred conditions suggests the intermediacy of a gel phase that precedes crystallization, consistent with previous reports.^37^

Upon heating the mixture for several hours, the nanocrystals precipitate from solution and are isolated by centrifugation. The precipitate is then washed with DMF and water prior to functionalization with oleic acid and oleylamine followed by precipitation using methyl acetate and centrifugation (Scheme 1). The functionalized nanoMOFs disperse in toluene and are separated from insoluble material in a final centrifugation step (8000 rpm, 10 minutes). Oleate/oleylamine terminated nanocrystals prepared in this way remain colloidally stable for >3 months without sedimentation (Fig. S9†). However, additional rounds of dispersal in toluene, precipitation with methyl acetate, and centrifugation reduced the ligand density and reduced the dispersibility (see ESI†).

The ratio of oleate, oleylamine, linker, and acetate in the nanocrystals was measured by digesting a known mass of the sample in D_2_SO_4_/DMSO-d_6_ and recording the NMR spectrum in the presence of an internal standard (see detailed discussion below and ESI†). Similar quantities of oleate and olelyamine are observed and suggest mixed coordination as depicted in Scheme 1. The coordination of amine ligands to Zr_6_O_8_H_4_(acrylate)_12_ has been described.^38^

Ligand exchange was conducted by stirring the toluene dispersion of nanocrystals with octadecylphosphonic acid (∼1 equiv. per surface amine and oleate) at 60 °C, followed by two cycles of precipitation, centrifugation, and dispersion using toluene and methyl acetate. Greater amounts of octadecylphosphonic acid caused dissolution of the framework, which may be related to decomposition of the Z_6_O_8_ nodal cluster.^39^ The presence of bound oleylamine reduces the acidity of the mixture and is sufficiently basic to deprotonate monohydrogen phosphonate ions, albeit in aqueous solution.^40–42^ Coordination to the Lewis acidic zirconium only increases the acidity and favors the formation of the dibasic phosphonate/ammonium ion pair proposed in Scheme 1 and similar to motifs found on colloidal quantum dots.^43^ Unlike the oleylamine/oleate terminated crystals, the ODPA terminated crystals remain colloidally stable after three washes.

### TEM & light scattering

**Fig. 2:**
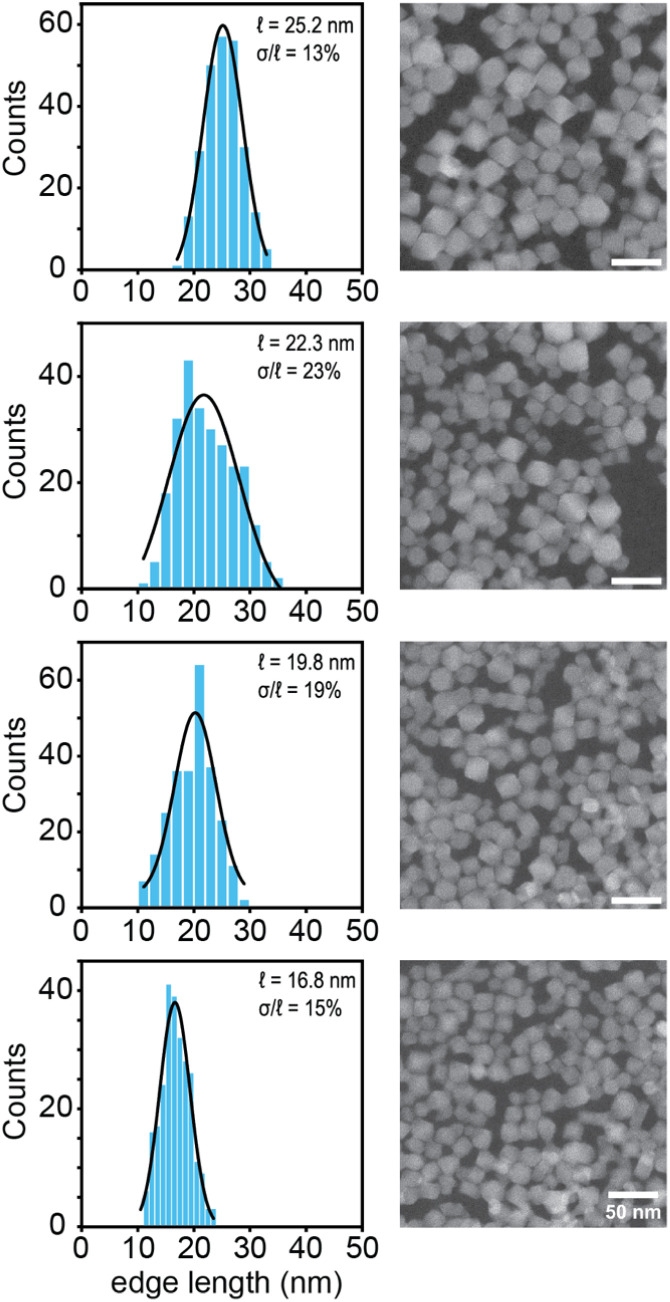
Histograms based on the edge length of ODPA-treated particles measured along adjacent vertices in the STEM images (inset shows the Gaussian fit). One representative image is shown alongside each histogram (scale bars = 50 nm).

Nanocrystals were studied at each stage of the synthesis and ligand exchange procedure using scanning transmission electron microscopy (STEM). Octahedral shapes from 16.8–25.2 nm are consistently observed provided that the crude products are washed with water. Larger and smaller sizes were also obtained by further adjusting the linker concentration, but suffered from interparticle fusion at smaller sizes and poor dispersibility at larger sizes. Histograms of the octahedron edge lengths measured using TEM are plotted in Fig. 2. Relatively narrow polydispersities are observed from 13% to 23% (Fig. 2).

Exchange of the oleate ligands for ODPA increased the separation between particles and helped eliminate some of the aggregation visible in the STEM images. Hydrodynamic diameters (∼25 nm), as measured using dynamic light scattering (DLS), are in good agreement with the dimensions determined using STEM. The hydrodynamic diameters of ODPA terminated crystals are 10–15 nm lower than the oleate and oleylamine functionalized nanocrystals (Fig. S10†). Hence, our TEM and DLS results support the conclusion that ODPA treatment decreases the degree of nanocrystal aggregation and enhances the dispersibility.

### NMR spectroscopy

^1^H liquids NMR spectra of MOF nanocrystals displayed broad signals that are assigned to linkers and surface bound surfactant ligands.^32^ The terephthalate linker signal is broader than the signals from ligands, presumably because of increased tumbling of the aliphatic ligand chain end and/or the dynamic exchange of free and bound ligands on the NMR timescale.^44–46^ The linker signal systematically and slightly broadens with increasing nanocrystal size, which suggests the spectral lineshape is dominated by nanocrystal tumbling and is not significantly affected by exchange.

**Fig. 3:**
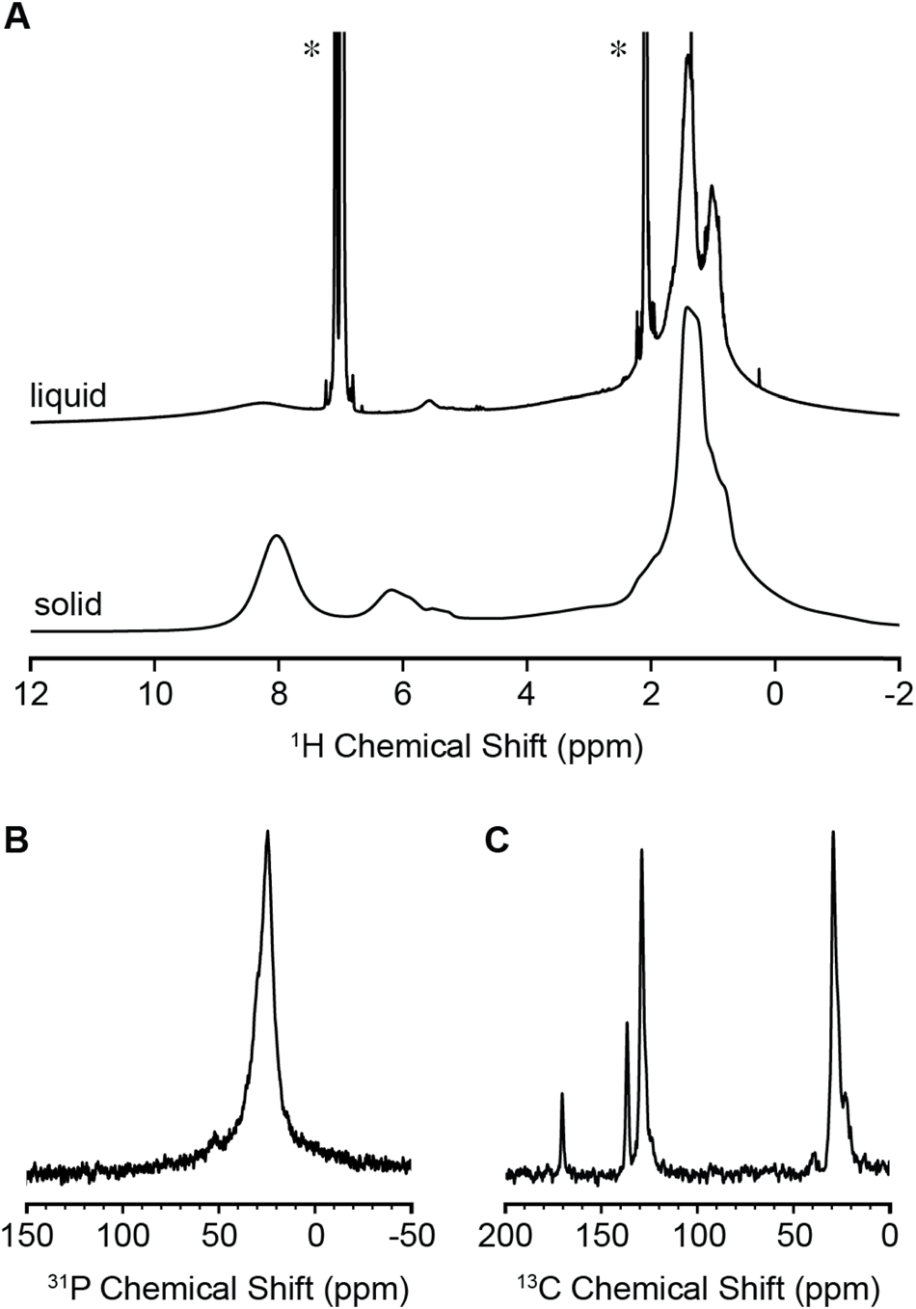
NMR spectra of 19.8 nm ODPA-capped MOFs. (A) Liquid phase (top) and solid state (bottom) ^1^H NMR spectra. Asterisk (*) denotes residual protonated solvent signals. (B) ^31^P solution phase NMR spectrum (50 Hz apodization applied). (C) ^13^C solid-state NMR spectrum.

Magic angle spinning (MAS) solid-state NMR (SSNMR) spectra are compared with the liquids NMR spectrum in Fig. 3. SSNMR spectra displayed significantly narrower linker signals than liquid spectra, which supports the conclusion that the NMR linewidth in the liquids spectrum is broadened by slow tumbling. However, the overall spectral lineshapes are similar. Both measurements support a relatively consistent and controlled ratio of ligands and linkers that is the result of our optimized synthesis and isolation procedure.

ODPA terminated nanocrystals display more isotropic signals, and narrower linker resonances (*δ* = 8–9 ppm) than oleate terminated crystals (Fig. S21†), that can be explained by their improved tumbling and lower degree of aggregation. At the same time, the sharp signals of free ligands are absent, which may be the result of a higher binding constant of the phosphonate headgroup and the tighter association of the ammonium ion as compared to the oleylamine ligand. The high affinity of phosphonic acids for early metal oxides is well established.^47–49^ Liquid phase ^31^P NMR spectra of the ODPA terminated particles display a broad peak centered at *δ* = 24.7 ppm with a full width at half maximum of 1600–2000 Hz, depending on particle size (Fig. 3 and S23†). This chemical shift and linewidth are similar to those reported for phosphonate terminated quantum dots.^50^

### Chemical formula determination

The chemical formula of the nanocrystals was measured using a combination of thermal gravimetric analysis (TGA) and NMR spectroscopy. The ratio of ligands and linkers and modulators was determined following quantitative digestion in a mixture of D_2_SO_4_ and DMSO-d_6_ under conditions that do not degrade the linker or ligand structures. (High concentrations of D_2_SO_4_ degraded the oleyl chain and caused H/D exchange.)

In the presence of an internal standard the ^1^H NMR spectra of the digestion solution allows precise determination of the linker and ligand mole percentages, including signals from oleylammonium and acetic acid. The mass percentage of zirconium (in the form of ZrO_2_) is measured by thermal gravimetric analysis (TGA) at temperatures up to 800 °C,^34,51^ from which the mole percentage of [Zr_6_(O)_4_(OH)_4_]^12+^ can be calculated. These results are compared with the ligand mole percentages determined by acid digestion and liquids NMR spectroscopy. However, this method underestimates the amount of ODPA, which has low solubility in D_2_SO_4_/DMSO-d_6_ (see ESI†). Hence, the ODPA content was determined from the ^1^H SSNMR spectrum by subtracting the contribution of acetate and oleylammonium ligands (determined following digestion) from the total aliphatic signal (see Section S2 of ESI†).

The mass percentages of linkers, ligands, acetate, and [Zr_6_(O)_4_(OH)_4_]^12+^ are shown in Table 1. The mass balance results indicate that the combined TGA/NMR approach is self-consistent. Interestingly, incorporation of modulators is relatively low (10 mol%), when compared to the ratio of acetic acid and terephthalic acid in the synthesis mixture (32 : 1 to 73 : 1).^52^ Although surface hydroxide may be present, the FT-IR spectrum of nanocrystals prior to and after functionalization with ODPA has relatively weak signals in the *v*(O–H) region. Collectively, these results are consistent with a relatively pure sample of our octahedral nanocrystal model.

**Table 1:** Mass percentage and ligand density of MOF nanocrystals following exchange with ODPA

*D* a (nm)	Zr_6_O_8_^b^ (%)	Linker^c^ (%)	R′-NH_*x*_^c^ (%)	ODPA^d^ (%)	Acetate^c^ (%)	Toluene^c^ (%)	Total (%)	R′-NH_*x*_^c^^,^^e^ (nm^−2^)	ODPA^d^^,^^e^ (nm^−2^)	Total (nm^−2^)
22.8(2.1)	30.8	32.2(3.2)	10.5(1.0)	17.5(1.8)	3.5(0.3)	4.5(0.5)	99(10)	1.1(0.2)	1.5(0.2)	2.6(0.4)
27.0(2.1)	31.1	31.5(3.1)	9.3(0.9)	19.8(2.0)	3.2(0.3)	5.2(0.5)	100(10)	1.1(0.2)	1.9(0.3)	3.0(0.4)
31.1(2.1)	31.5	34.4(3.4)	9.4(0.9)	18.4(1.8)	3.6(0.4)	6.4(0.6)	104(10)	1.2(0.2)	1.9(0.3)	3.2(0.4)
35.3(2.1)	31.3	29.2(2.9)	6.8(0.7)	17.2(1.7)	2.7(0.3)	1.6(0.2)	89(9)	1.0(0.1)	2.0(0.3)	3.0(0.4)

a The error in *D* is assumed to be equal to the dimension of a single UiO-66 unit cell.

b Determined using TGA. Uncertainty in TGA mass measurement is 10^−6^ mg, hence the error is negligible.

c Determined from quantitative digestion and liquids ^1^H NMR (see Section S2 of ESI). Error in values determined from NMR peak integration is assumed to be 10%.

d From SSNMR integration of aliphatic signal (see Section S2 of ESI).

e Uncertainty in the ligand density was calculated from 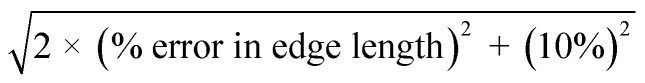, taken from the uncertainty in the TEM measurement of the edge length 
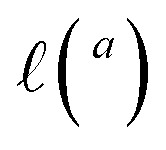
, and the NMR measurements (^*c*^) assuming the surface area of the nanocrystals is that of an octahedron 
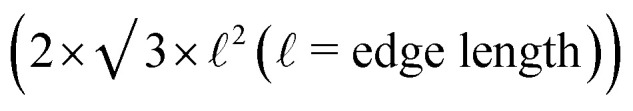
.

The areal density of oleyl and ODPA chains reaches 2.6–3.2 nm^−2^, which is similar to the density of carboxylates on colloidal quantum dots,^53^ and well below the density of crystalline *n*-alkanes (4.9 nm^−2^) (see ESI†). This quantity is approximately ∼2× the theoretical density of surface carboxylates on the (111) surface (1.61 nm^−2^) and consistent with the proposed oleylammonium octadecylphosphonate structure shown in Scheme 1. The relatively high density of chains helps explain the persistent colloidal stability of the final nanocrystals.

However, the measured density of ODPA is greater than oleylamine and slightly greater than the density of carboxylate binding sites. A portion of the ligands may exist in a monohydrogen phosphonate form, rather than the dibasic form with associated ammonium cations. Alternatively, partial decomposition of the nodal cluster may lead to other forms. In any case, the similarity of these data and the theoretical octahedral nanocrystal supports a picture where oleate, oleylammonium, and octadecylphosphonate ligands are bound to surface coordination sites, while modulators and linkers are associated with the interior of the nanocrystal.

### Gas uptake

With knowledge of their empirical formula and ligand coverage, we investigated whether their gas uptake capacity is influenced by their size and surface ligand environment. We measured the CO_2_ and N_2_ gas uptake capacities of MOF nanocrystals before and after functionalization with ODPA (Table 2). Oleate-terminated nanocrystals exhibit an order of magnitude lower capacity compared to bulk UiO-66 on a per gram basis.^54,55^ By subtracting the mass of the ligands from the total mass, we estimate the intrinsic gas uptake capacity of the nanocrystalline MOF. This suggests a capacity of ∼ half (N_2_) or ∼ a quarter (CO_2_) of the bulk capacity (Fig S17†). Interestingly, functionalization with ODPA increases the capacity two-fold, which suggests that the capacity is influenced by the degree of interparticle aggregation. We note that the gas uptake values are known to be sensitive to the presence of modulator related defects.^34^ As the nanocrystal size increases, the capacity increases, which is consistent with a greater capacity of the bulk compared to the nanocrystal surface.

**Table 2:** Change in gas absorption capacity upon ODPA treatment

Edge length (nm)	*D* (nm)	CO_2_ capture oleate^a^ (mmol g^−1^)	CO_2_ capture ODPA^a^ (mmol g^−1^)	Surface area oleate (m^2^ g^−1^)	Surface area ODPA (m^2^ g^−1^)
16.8	22.8	0.157	0.311	152	369
19.8	27.0	0.157	0.360	140	379
22.3	31.1	0.162	0.378	148	392
25.2	35.3	0.171	0.351	81	nd^b^
Bulk		1.786		1067	

a CO_2_ uptake capacity was measured using TGA at 25 °C, under 1 atmosphere of CO_2_.

b The yield of the 25.2 nm ODPA capped MOF synthesis was insufficient for a BET measurement.

## Conclusions

Here, we demonstrate that MOF nanocrystals may be dispersed in nonpolar solvents using oleate, oleylamine, and octadecylphosphonate ligands. The chemical formulas of these nanocrystals are consistent with the size dependent theoretical formula of an octahedron on the nanometer length scale with surface ligand areal densities near the theoretical value of carboxylate densities on the (111) surface. The relatively high density of ligands enables indefinitely stable colloidal dispersions that can be analysed using liquids NMR spectroscopy. The gas uptake capacity of such particles increased upon improving the dispersibility (*i.e.* reducing the aggregation) following ligand exchange. The improvements in the gas uptake imply that further exploration of the surface coordination chemistry could lead to even higher gas absorption and improved MOF-polymer composite materials.

## Data availability

Experimental data is available in the ESI online.†

## Author contributions

The manuscript was written through contributions of all authors.

## Conflicts of interest

There are no conflicts to declare.

## Supplementary Material

SC-OLF-D4SC06528J-s001
