# Supplementary material for: Steric stabilization of colloidal UiO-66 nanocrystals with oleylammonium octadecylphosphonate
Source: Chem Sci. 2024 Dec 2;16(2):933–8. doi: 10.1039/d4sc06528j (PMC11626757; doi:10.1039/d4sc06528j)
Supplement: SC-016-D4SC06528J-s001 [file SC-016-D4SC06528J-s001.pdf]

Supplementary Information (SI) for Chemical Science.
This journal is © The Royal Society of Chemistry 2024

Supplementary Information for

Steric Stabilization of Colloidal UiO-66 Nanocrystals with Oleylammonium Octadecylphosphonate

Sungho V. Park,[1] Lakshmi Bhai,[2] Gahyun Annie Lee,[2,3] Ah-Hyung A. Park,[2,3,4] Lauren E. Marbella,[2] and Jonathan S. Owen[1]*

[1] Department of Chemistry, Columbia University, New York, NY 10027, United States

[2] Department of Chemical Engineering, Columbia University, New York, New York 10027, United States

[3] Lenfest Center for Sustainable Energy, Columbia University, New York, NY 10027, United States

[4] Department of Earth and Environmental Engineering, Columbia University, New York, New York 10027, United States

* To whom correspondence may be addressed: jso2115@columbia.edu

Sections:

Section S1: Experimental details S2

Section S2: MOF chemical formula determination method S4

Section S3: Supplementary figures S8

Section S4: BET, TGA, and CO_2 capture analysis S15

Section S5: Modelling S19

Section S6: Solution phase NMR spectra S21

Section S7: References S26

SECTION S1: EXPERIMENTAL DETAILS

General Considerations. All operations were conducted in air unless otherwise specified. N,N-Dimethylformamide (ACS reagent, ≥99.8%), acetic acid (glacial, ACS reagent, ≥99.7%), and oleic acid (≥99%) were purchased from Sigma-Aldrich. Zirconyl chloride octahydrate (98%) and terephthalic acid (99+%) were purchased from Acros Organics. Oleylamine (technical grade, 70%) was purchased from Sigma-Aldrich and fractionally distilled according to a literature procedure.[1] Toluene-d_8 (99+ atom % D) was purchased from Thermo Scientific and stored over molecular sieves. Toluene (ACS reagent, ≥99.5%) and methyl acetate (ReagentPlus®, 99%) were purchased from Sigma-Aldrich and stored over molecular sieves. All ^1H NMR spectra were referenced to residual protiated solvent signals (2.08 ppm for toluene-d_7, 2.50 ppm for DMSO-d_5, and 2.92 ppm for DMF-d_6).[2] All ^{31}P NMR spectra were referenced to the corresponding ^1H spectra using absolute referencing.[3, 4]

Synthesis of the MOF nanocrystals. In a vial, terephthalic acid (1.92 g for 16.8 nm MOFs, 1.44 g for 19.8 nm MOFs, 1.08 g for 22.3 nm MOFs, and 840 mg for 25.2 nm MOFs) was loaded, and an appropriate amount of DMF was added such that the concentration of terephthalic acid would equal 0.3 M. The mixture was sonicated until it appeared completely homogeneous (~ 7 minutes). In a 250 mL 3-neck flask, zirconyl chloride (630 mg) was added, and DMF was added such that the total amount of DMF in the entire reaction mixture would equal 120 mL. Afterwards, the linker solution and 21 mL acetic acid was added via funnel, and the reaction mixture was placed in a mantle and heated to 100 °C using a J-KEM 210 temperature controller with a ramp rate of 300 °C / hour with stirring at 450 rpm under Ar flow. After 18 hours, the reaction vessel was lifted from the mantle and stirring was stopped. The reaction mixture was allowed to cool to room temperature for one hour, after which it was divided into 6 centrifuge tubes and centrifuged at 11000 rpm for 20 minutes. The resulting precipitate was washed once with DMF and once with DI water.

Functionalization of the MOF nanocrystals with oleic acid and oleylamine. This was conducted immediately after the previous procedure. Oleic acid (1.7 mL) was added to each centrifuge tube, followed by 5 mL of toluene. The centrifuge tubes were manually shaken for 20 seconds, after which oleylamine (1.8 mL) was added and the centrifuge tubes were manually shaken for an additional 3 minutes each. Afterwards, 25 mL methyl acetate was added to each tube to crash out the nanocrystals, and the resulting mixtures were centrifuged at 10000 rpm for 10 minutes. The supernatant was decanted, and the remaining precipitate was redispersed in 3 mL toluene, after which 15 mL MeOAc was added. The resulting mixtures were centrifuged once more at 10000 rpm for 10 minutes. Afterwards, the precipitate was redispersed in 8 mL toluene and centrifuged at 8000 rpm for 10 minutes. The supernatant was collected and filtered through a 0.2 um filter before drying under vacuum overnight to give the final product. Yield: 463.5 mg (16.8 nm MOF), 391.9 mg (19.8 nm MOF), 406.2 mg (22.3 nm MOF), 293.3 mg (25.2 nm MOF).

Treatment with octadecylphosphonic acid (ODPA). To the oleate capped nanocrystals obtained from the previous procedure, ODPA and toluene were added (see amounts below) in a 100 mL round bottom flask and the resulting mixture was sonicated for 10 minutes. Afterwards, the reaction mixture was stirred at 60 °C for 2 hours in an oil bath with stirring at 300 rpm under Ar flow (for the 25.2 nm particles, the reaction mixture was stirred at room temperature for 13 hours). The reaction mixture was then removed from the oil bath and stirring was stopped. After cooling to room temperature, the reaction mixture was washed with 1:4 toluene:methyl acetate twice (9000 rpm, 10 minutes). The final product was redispersed in toluene (same amount as below) and centrifuged at 8000 rpm for 10 minutes. The supernatant was collected and filtered through a 0.2 um filter before drying under vacuum overnight to give the final product.

	16.8 nm	19.8 nm	22.3 nm	25.2 nm
Starting MOF	463.5 mg	391.9 mg	406.2 mg	293.3 mg
ODPA	83.1 mg	72.0 mg	62.5 mg	52.7 mg
Toluene	29 mL	24 mL	25 mL	37 mL
Yield	213.5 mg	193.1 mg	213.2 mg	80.2 mg

Acid digestion. To prepare the stock solution of internal standard, 10.3 mg of dimethyl sulfone was dissolved in 7.00 mL DMSO-d_6 and the mixture was sonicated for 10 minutes. In a 4 mL vial, >4 mg of MOF was measured. To the MOF was added 0.70 mL of internal standard stock solution and 5 drops of D_2SO_4. The resulting mixtures were sonicated for 45 minutes. The NMR samples were measured with a d1 = 26 seconds.

Percent mass recovered. Percent mass recovered was obtained by dividing the mass of MOF product obtained by the theoretical mass. Theoretical mass was calculated with 100% exterior surface coverage with oleate or octadecylphosphonate ligands and 100% interior surface coverage with acetate:linker ratio determined from digestion NMR.

	16.8 nm	19.8 nm	22.3 nm	25.2 nm
Mass zirconyl chloride	630 mg	630 mg	630 mg	630 mg
Mass terephthalic acid	1920 mg	1440 mg	1080 mg	840 mg
Experimental mass of oleate capped MOF	463.5 mg	391.9 mg	406.2 mg	293.3 mg
Theoretical mass (100% oleate coverage)	629.1 mg	615.7 mg	604.7 mg	597.6 mg
**% mass recovered**	**74%**	**64%**	**67%**	**49%**
Experimental mass of ODPA capped MOF	213.5 mg	193.1 mg	213.2 mg	80.2 mg
Theoretical mass (100% ODPA coverage)	654.4 mg	637.4 mg	623.7 mg	614.5 mg
**% mass recovered**	**33%**	**30%**	**34%**	**13%**

Final yield determination. The overall yield (over three steps) was determined from the TGA data, which gives the % mass of Zr in the final, ODPA-treated MOF material.

	16.8 nm	19.8 nm	22.3 nm	25.2 nm
Mass yield	213.5 mg	193.1 mg	213.2 mg	80.2 mg
Mass % from node	30.80	31.06	31.46	31.31
Limiting reagent	630 mg zirconyl chloride	630 mg zirconyl chloride	630 mg zirconyl chloride	630 mg zirconyl chloride
**Finalized % yield**	**30%**	**27%**	**30%**	**11%**

Dynamic light scattering (DLS) measurements. As a small degree of aggregation was visible in the STEM images, we used the sparse Bayesian learning (SBL) algorithm for data analysis; indeed, the cumulants method gave a systematic overestimation of DLS-derived sizes for all samples. Based on the number distribution, which is the most accurate representation of size, ODPA treatment leads to a decrease of 10-15 nm in particle diameter.

SECTION S2: MOF CHEMICAL FORMULA DETERMINATION METHOD

TGA for Zr_6O_8 content determination. A sample of ODPA capped MOFs (19.8 nm edge length) was loaded on a platinum pan. Under an O_2 flow (50 mL/min), the temperature was raised to 800 °C at a rate of 5 °C per minute. The starting mass was 7.79522 mg, and the end mass was 2.63447 mg. The material remaining at the end of the TGA sequence was assigned to 100% ZrO_2.

Conversion of ZrO_2 mass into mass of node ($[Zr_6O_4(OH)_4]^{12+}$): $2.63447\ \text{mg} \times \frac{679.37\frac{g}{mol}}{123.22\frac{g}{mol}\times 6} = 2.4208\ \text{mg}$.

Mass% of $[Zr_6O_4(OH)_4]^{12+}$ in MOF $= \frac{2.4208\ mg}{7.79522\ mg} \times 100\% = 31.056\%$.

Digestion NMR for ligand content determination. Dimethyl sulfone (10.3 mg) was dissolved in 7.00 mL of DMSO-d_6 and the mixture was sonicated for 10 minutes. In a 4 mL vial, 4.6 mg of ODPA capped MOFs (19.8 nm edge length) was measured. To the MOF was added 0.70 mL of the dimethyl sulfone stock solution and 5 drops of D_2SO_4. After the resulting mixture was sonicated for 45 minutes, its ^1H NMR spectrum was acquired with d1 = 26 seconds.

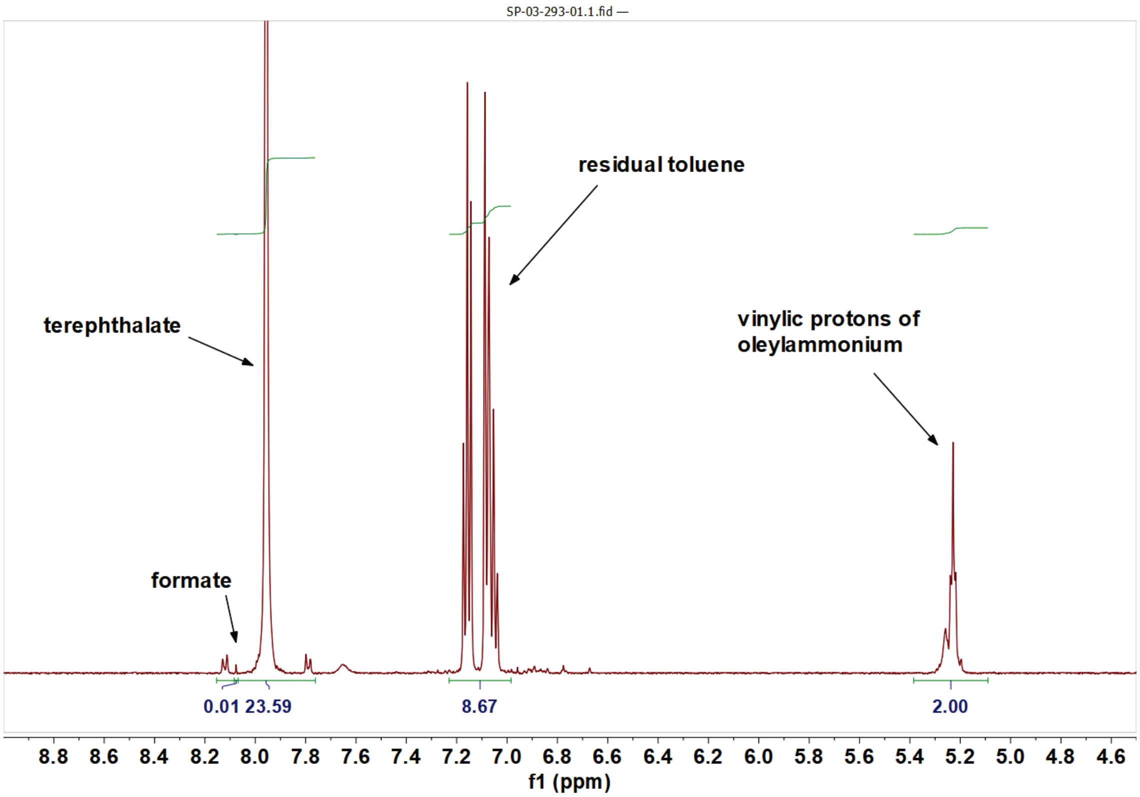

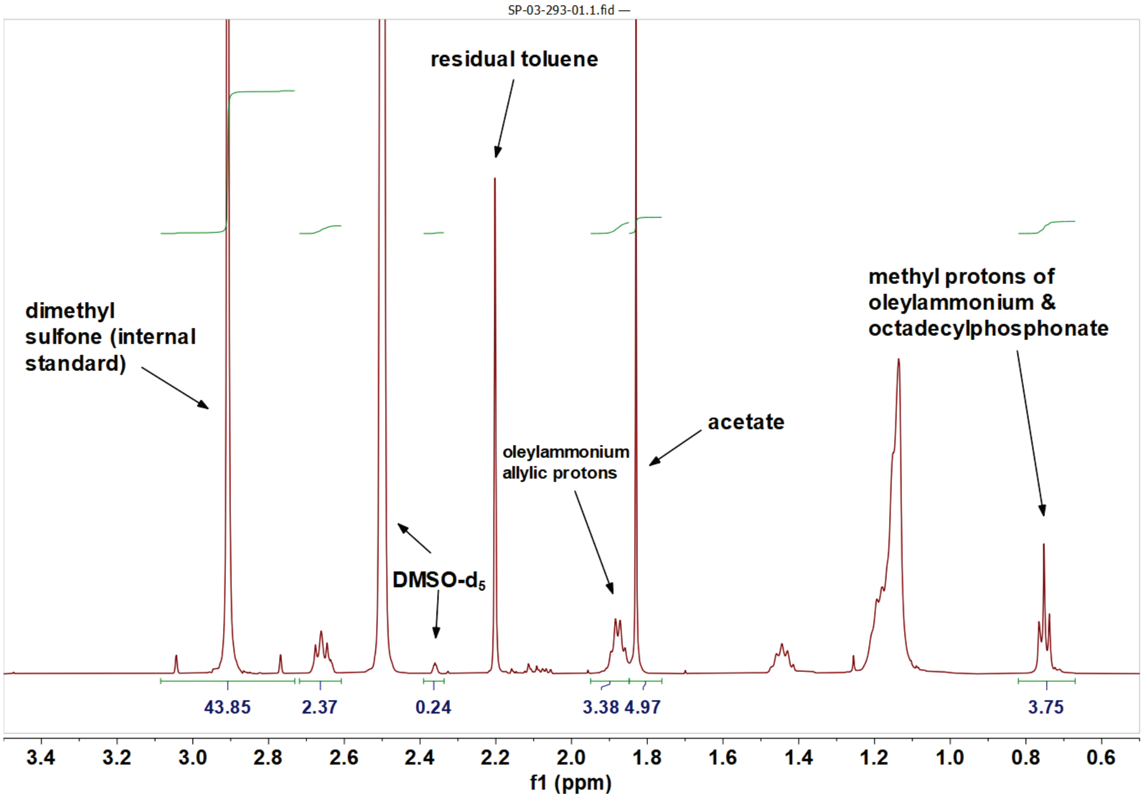

Figure S1. Annotated ^{1}H NMR spectrum of ODPA treated MOFs (19.8 nm edge length) after digestion with D_2SO_4/DMSO-d$_6$ with dimethyl sulfone as internal standard.

	Dimethyl sulfone (internal standard)	Linker	Oleylammonium	Acetate	Toluene
NMR peak integration value	43.85	23.58	2.37 - 0.24 = 2.13 (CH_2 signal α to the $-NH_3^+$)	4.97	8.67 (arom-H)
Moles in digestion sample	$\frac{\frac{10.3\,mg}{94.13\,g/mol}}{}\times\frac{0.70\,mL}{7.00\,mL} = 0.0109$ mmol	0.0109 mmol × $\frac{23.58/4H}{43.85/6H}$ = 0.00883 mmol	0.0109 mmol × $\frac{2.13/2H}{43.85/6H}$ = 0.00159 mmol	0.0109 mmol × $\frac{4.97/3H}{43.85/6H}$ = 0.00248 mmol	0.0109 mmol × $\frac{8.67/5H}{43.85/6H}$ = 0.00260 mmol
Mass in MOF sample	-	1.45 mg	0.428 mg	0.146 mg	0.239 mg

We have attempted to calculate ODPA content from the digestion NMR ^{1}H spectra, by subtracting the contribution from the oleylammonium to the methyl-CH_3 signal. However, this led to a severe underestimation of surface ODPA content, with oleylammonium:ODPA ratios ranging from 2.9 to 5.6, resulting in a charge-unbalanced MOF chemical formula. This can be attributed to the insolubility of ODPA in D_2SO_4/DMSO-d$_6$. Hence, solid state NMR was used to determine ODPA content.

Solid state NMR for ODPA content determination. An internal standard was not used in the ssNMR measurements; instead, the relative molar amounts of linker, oleylammonium and acetate determined from the digestion ^{1}H NMR measurement were used to deconvolute the aliphatic signal in the solids ^{1}H spectrum. The aliphatic signal, arising from the aliphatic protons in oleylammonium (33H), acetate (3H), and ODPA (37H), was integrated relative to the linker aromatic-H signal. After the contribution from acetate and oleylammonium ligands as determined from the digestion spectrum was subtracted, the remaining peak intensity was assigned to the aliphatic protons in the ODPA chain. This enabled calculation of the linker:ODPA molar ratio in the MOF sample.

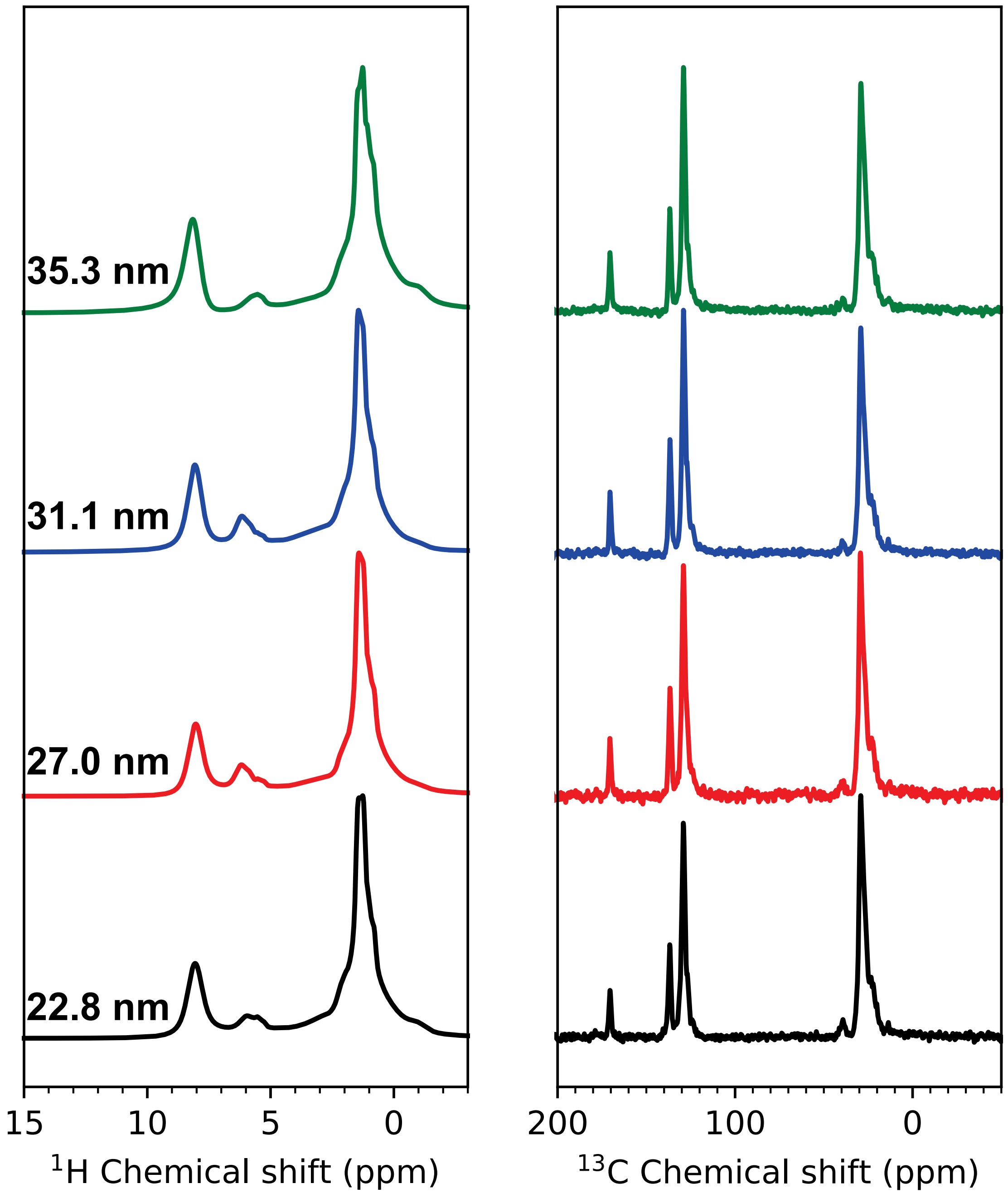

Figure S2. ^{1}H and ^{13}C solid state NMR spectra of ODPA treated MOFs (inset denotes body diagonal (D) of nanocrystal).

Error analysis. The error in D is assumed to be equal to the dimension of a single UiO-66 unit cell. Error in values determined from NMR peak integration is assumed to be 10%. Error propagation method was adapted from the literature.[5]

D (nm)	STEM edge size (nm)	Uncertainty for chain density
22.8(2.1)	16.8 ± 1.5	$\sqrt{(\frac{1.5}{16.8})^2 + (\frac{1.5}{16.8})^2 + 0.1^2} = 0.16$
27.0(2.1)	19.8 ± 1.5	$\sqrt{(\frac{1.5}{19.8})^2 + (\frac{1.5}{19.8})^2 + 0.1^2} = 0.14$
31.1(2.1)	22.3 ± 1.5	$\sqrt{(\frac{1.5}{22.3})^2 + (\frac{1.5}{22.3})^2 + 0.1^2} = 0.14$
35.3(2.1)	25.2 ± 1.5	$\sqrt{(\frac{1.5}{25.2})^2 + (\frac{1.5}{25.2})^2 + 0.1^2} = 0.13$

SECTION S3: SUPPLEMENTARY FIGURES

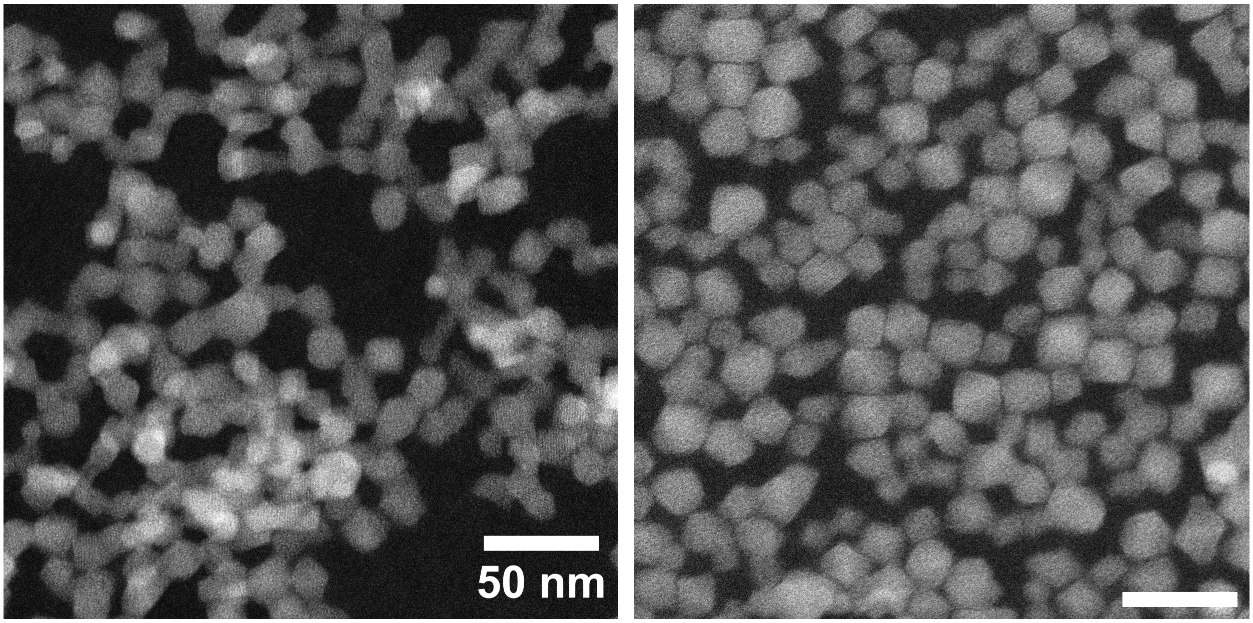

Figure S3. STEM images of 16.8 nm MOFs before (left) and after (right) ODPA treatment.

Acetic acid concentration	**2.6 M**	**3.1 M**	**3.6 M**	**4.1 M**
Synthesis **with stirring** (scale bar = 50 nm)	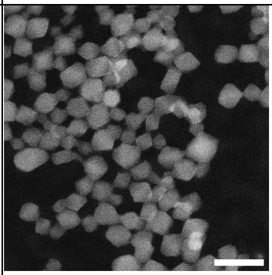	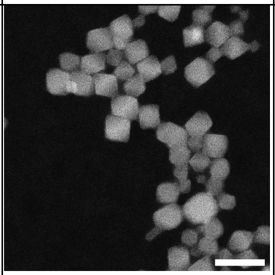	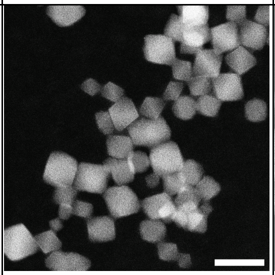	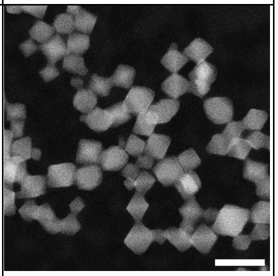
Synthesis **without stirring** (scale bar = 200 nm)	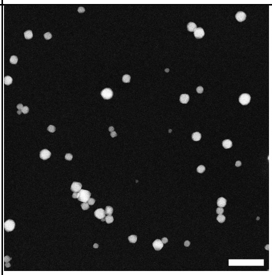	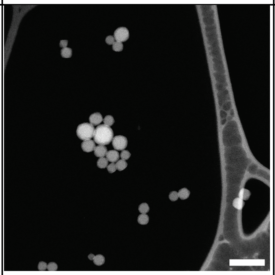	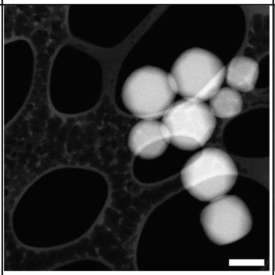	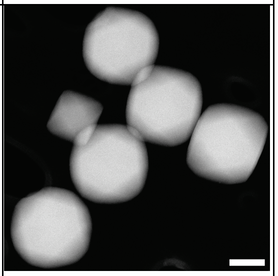

Figure S4. Effect of changing modulator (acetic acid) concentration on MOF particle size. Images are taken after functionalization with oleic acid and oleylamine.

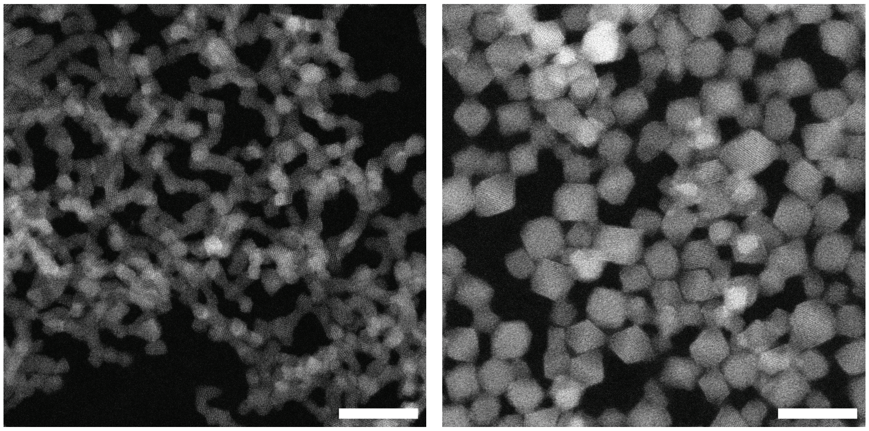

Figure S5. (Left) MOFs synthesized with the linker added as a solid. (Right) MOFs synthesized with the linker added as a homogeneous solution in DMF (scale bar = 50 nm).

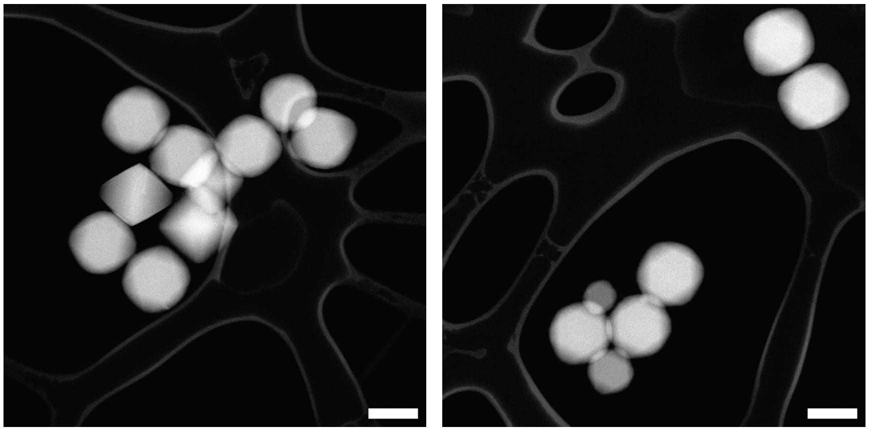

Figure S6. MOFs obtained upon sonication of zirconyl chloride in DMF prior to MOF synthesis (scale bar = 250 nm).

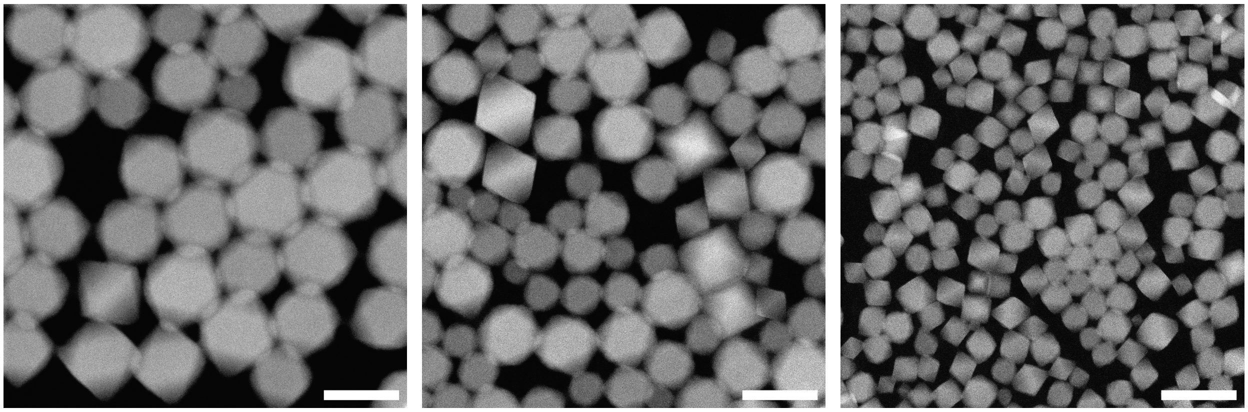

Figure S7. Effect of synthesis temperature on MOF nanocrystal size. MOFs synthesized at 80 °C, 90 °C, and 100 °C (scale bar = 100 nm).

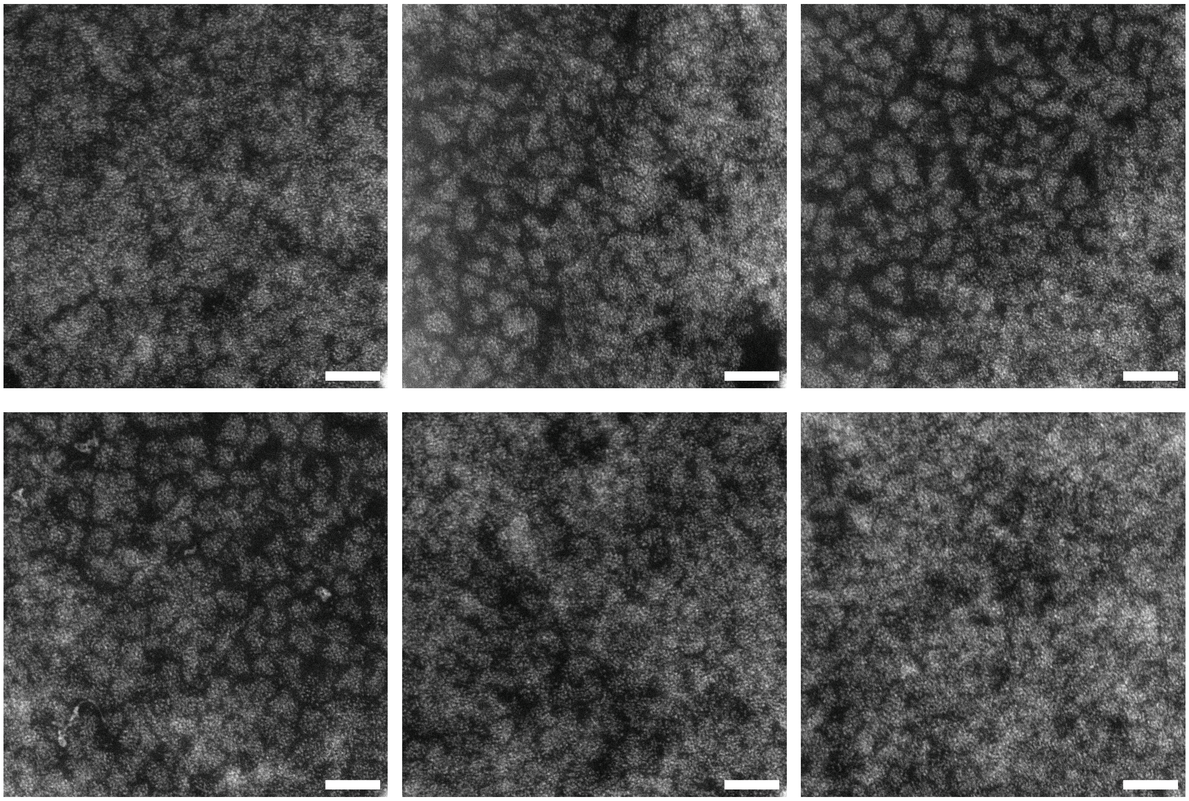

Figure S8. UiO-66 MOFs synthesized using oleic acid (instead of acetic acid) as modulator (scale bar = 25 nm).

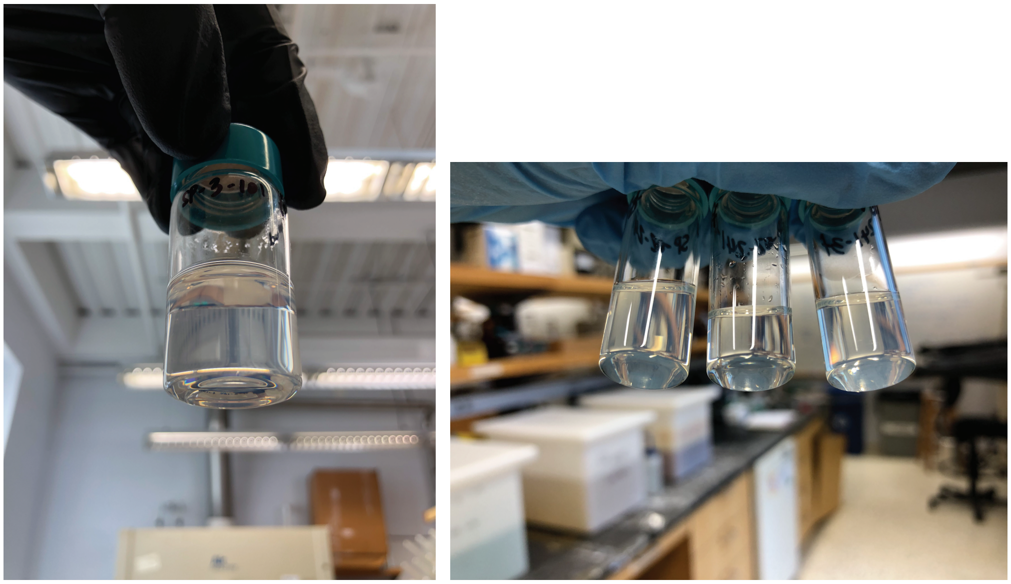

Figure S9. Photos of translucent toluene dispersions of oleate capped MOFs, which remain colloidally stable without sedimentation for >3 months.

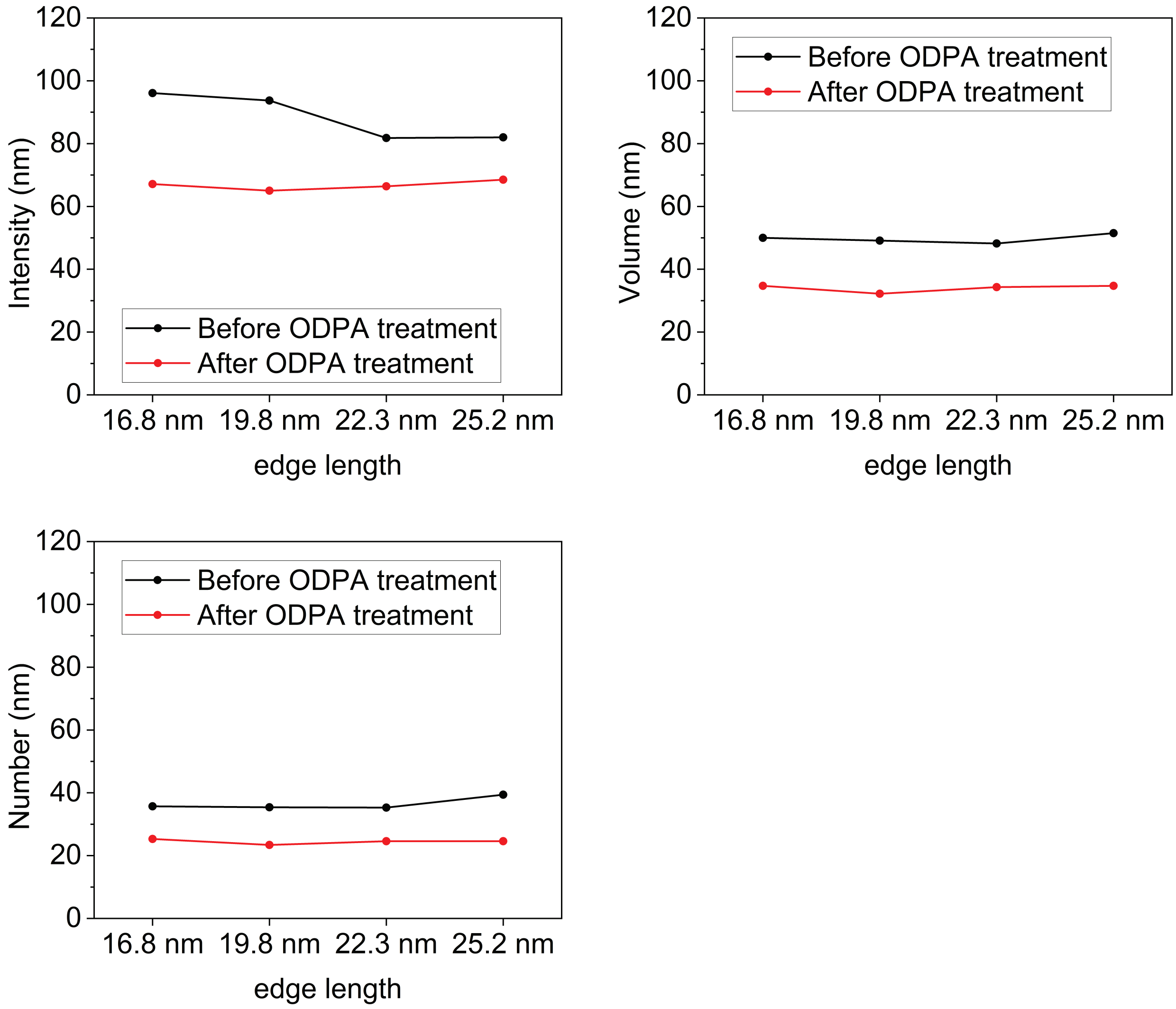

Figure S10. DLS data obtained for particles before and after ODPA treatment (processed using the SBL algorithm).

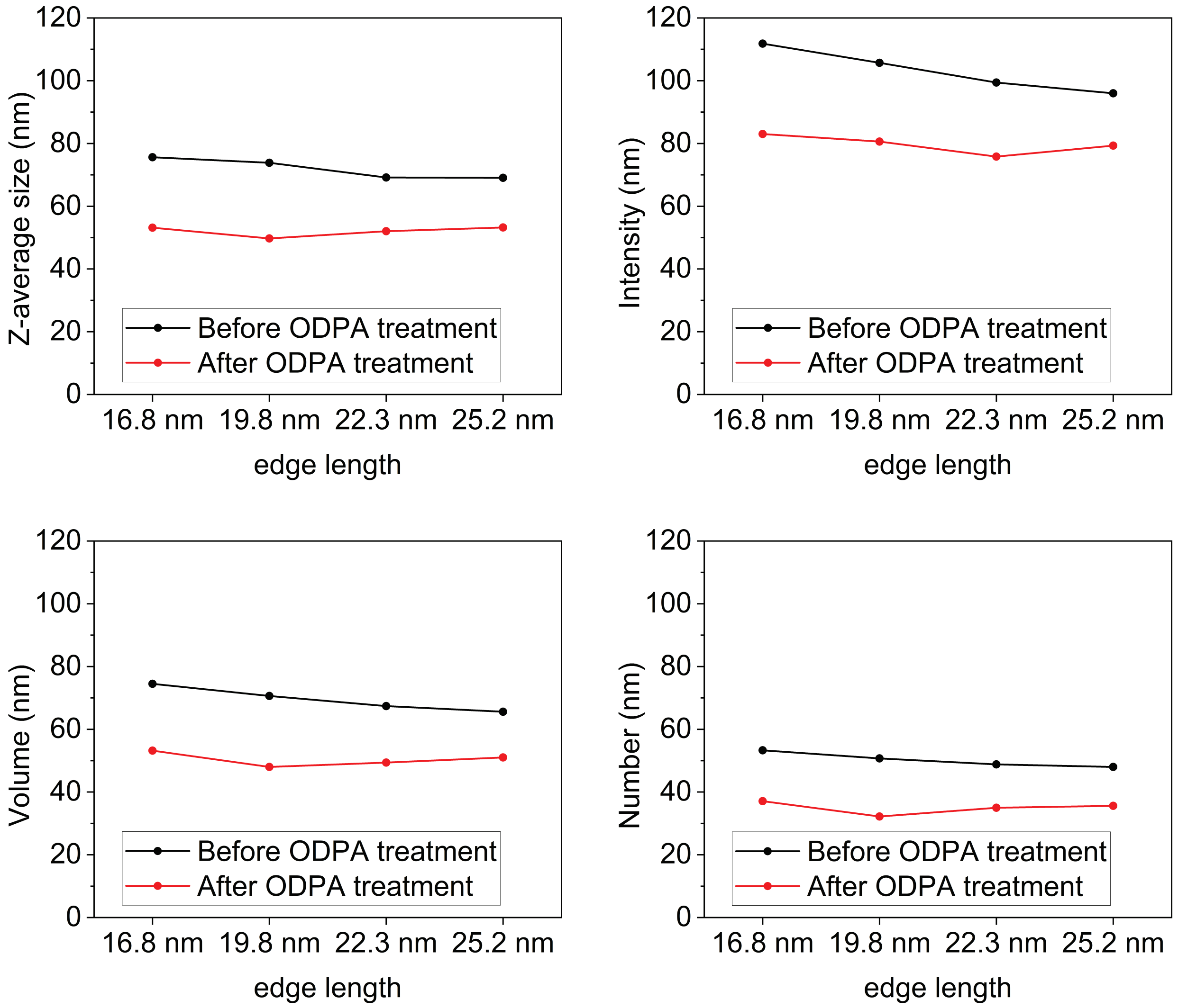

Figure S11. DLS data obtained for particles before and after ODPA treatment (processed using the cumulants method).

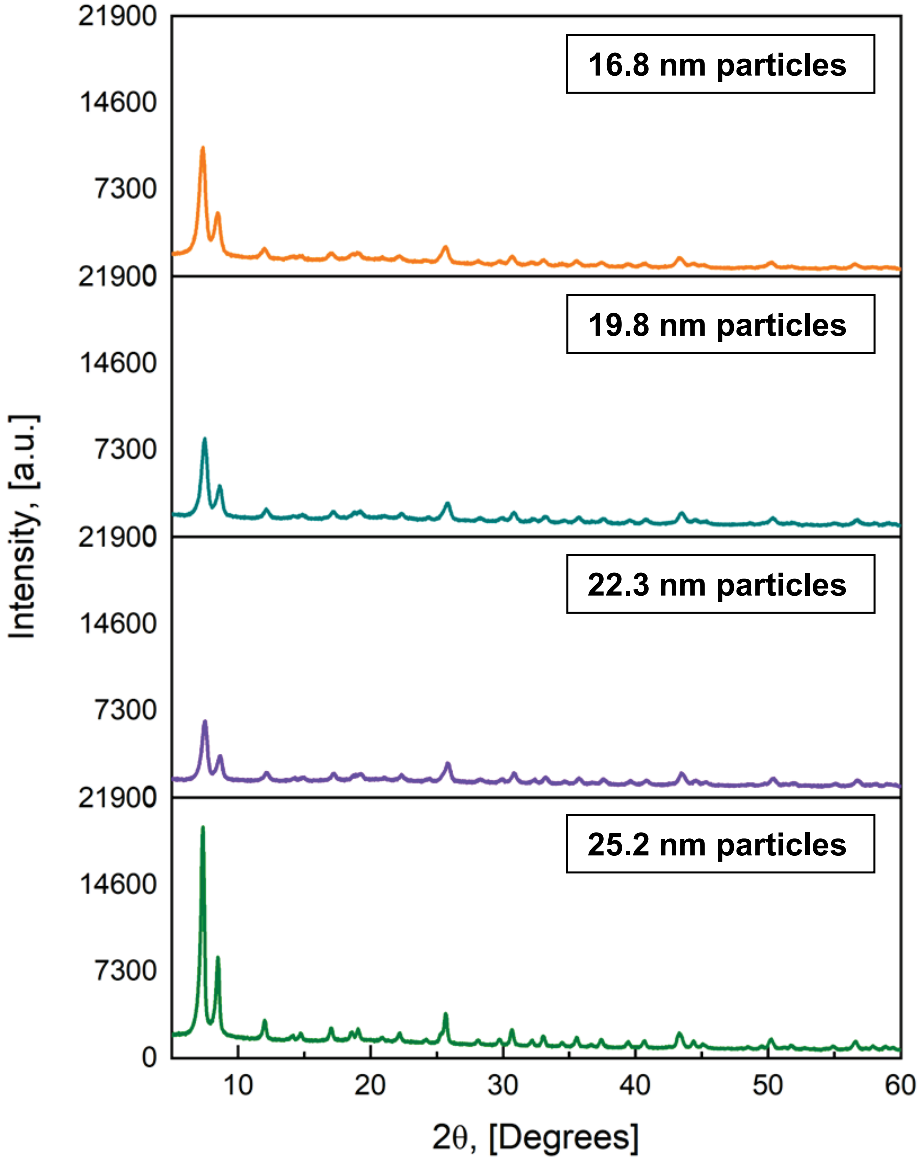

Figure S12. PXRD characterization of oleate capped MOFs.

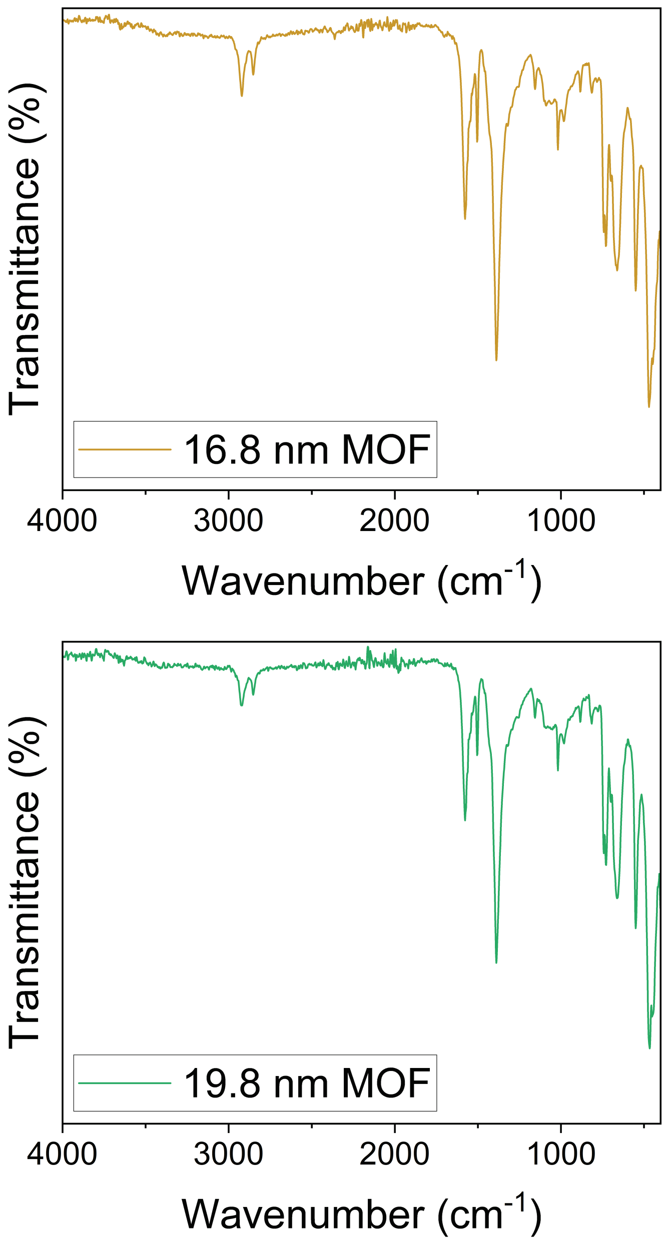

Figure S13. IR characterization of ODPA-treated MOFs.

SECTION S4: BET, TGA, and CO_2 capture analysis

- For BET analysis, all samples were activated under high vacuum at 100 °C for 14 hours.
- For CO_2 capture analysis, the ODPA-capped particles were activated under N_2 atmosphere at 150 °C for 1 hour in the TGA apparatus (100 °C for 1 hour for oleate-capped particles, due to sample stability concerns).

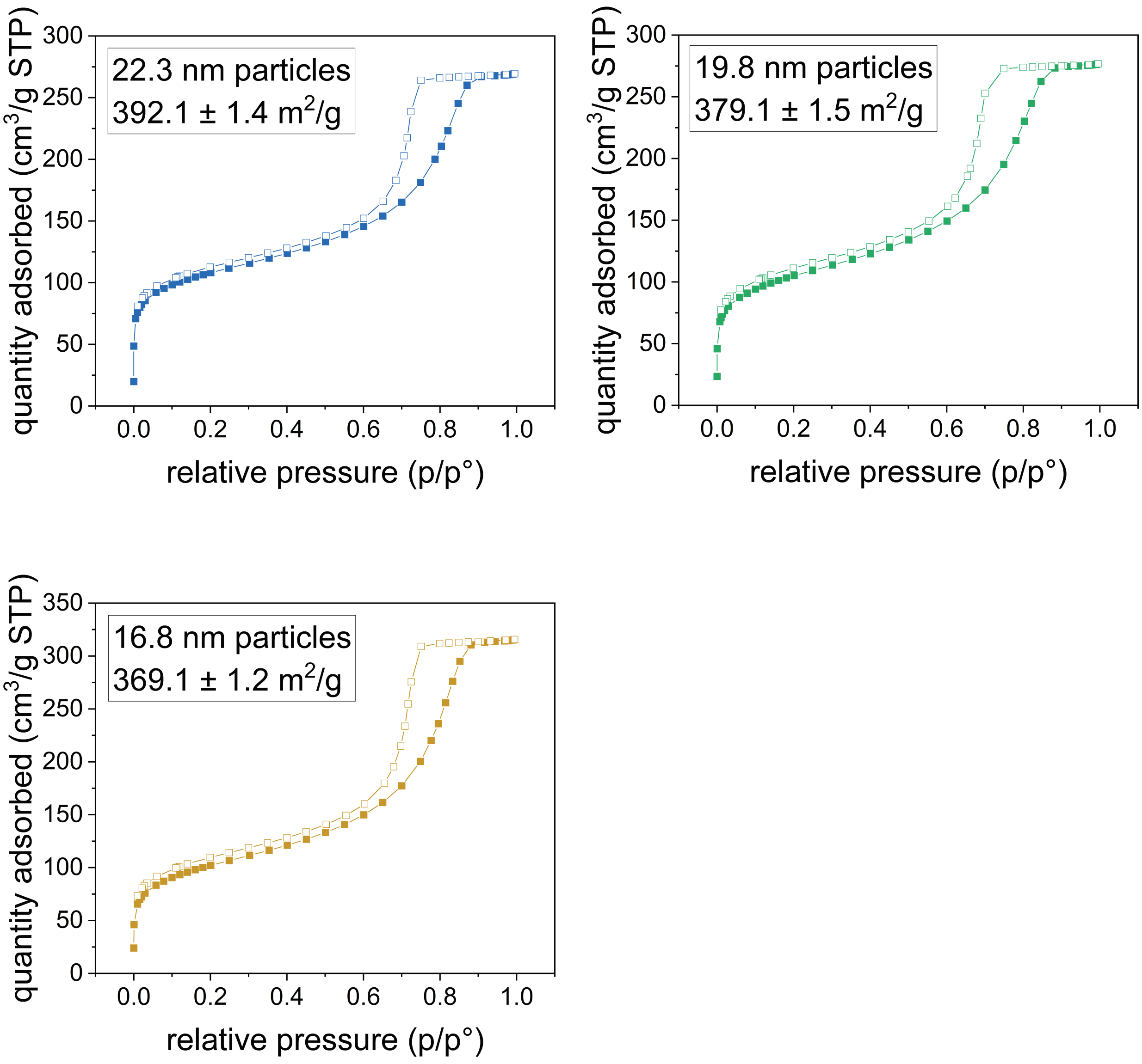

Figure S14. N_2 isotherms for ODPA-treated MOFs (inset describes particle size and BET surface area).

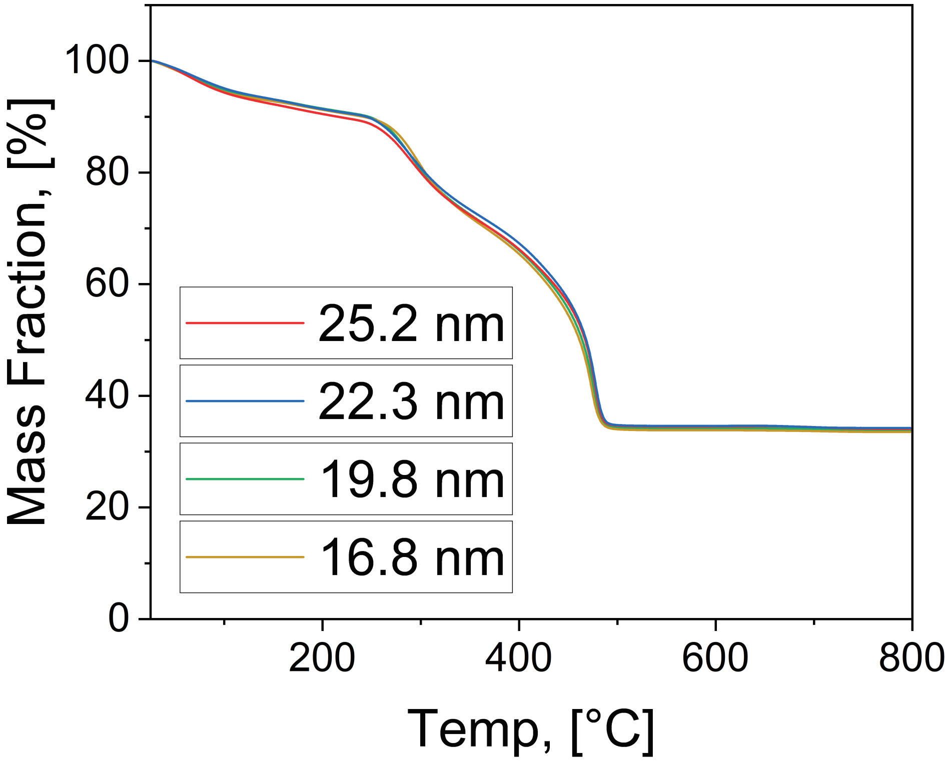

Figure S15. TGA traces for ODPA-treated MOFs.

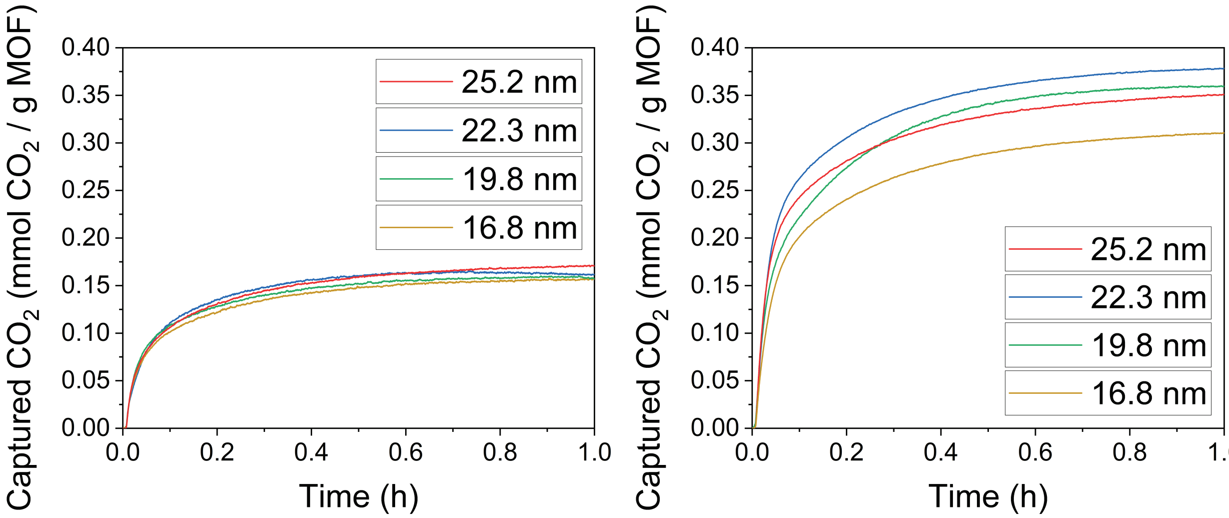

Figure S16. Effect of ODPA treatment on CO_2 absorption capacity. Oleate capped MOFs are shown on the left and ODPA treated MOFs are shown on the right.

Ligand-corrected gas uptake. Mass of the "MOF core" was defined as the sum of the masses of linker, Zr_6O_8, acetate, and formate. The CO_2 uptake capacities were corrected by dividing the measured capacities by the mass% of the MOF core relative to the original MOF mass. BET surface areas were corrected by dividing the measured surface areas by the mass% of the MOF core relative to the sum of the individual MOF component masses (see Table 1 in the main text).

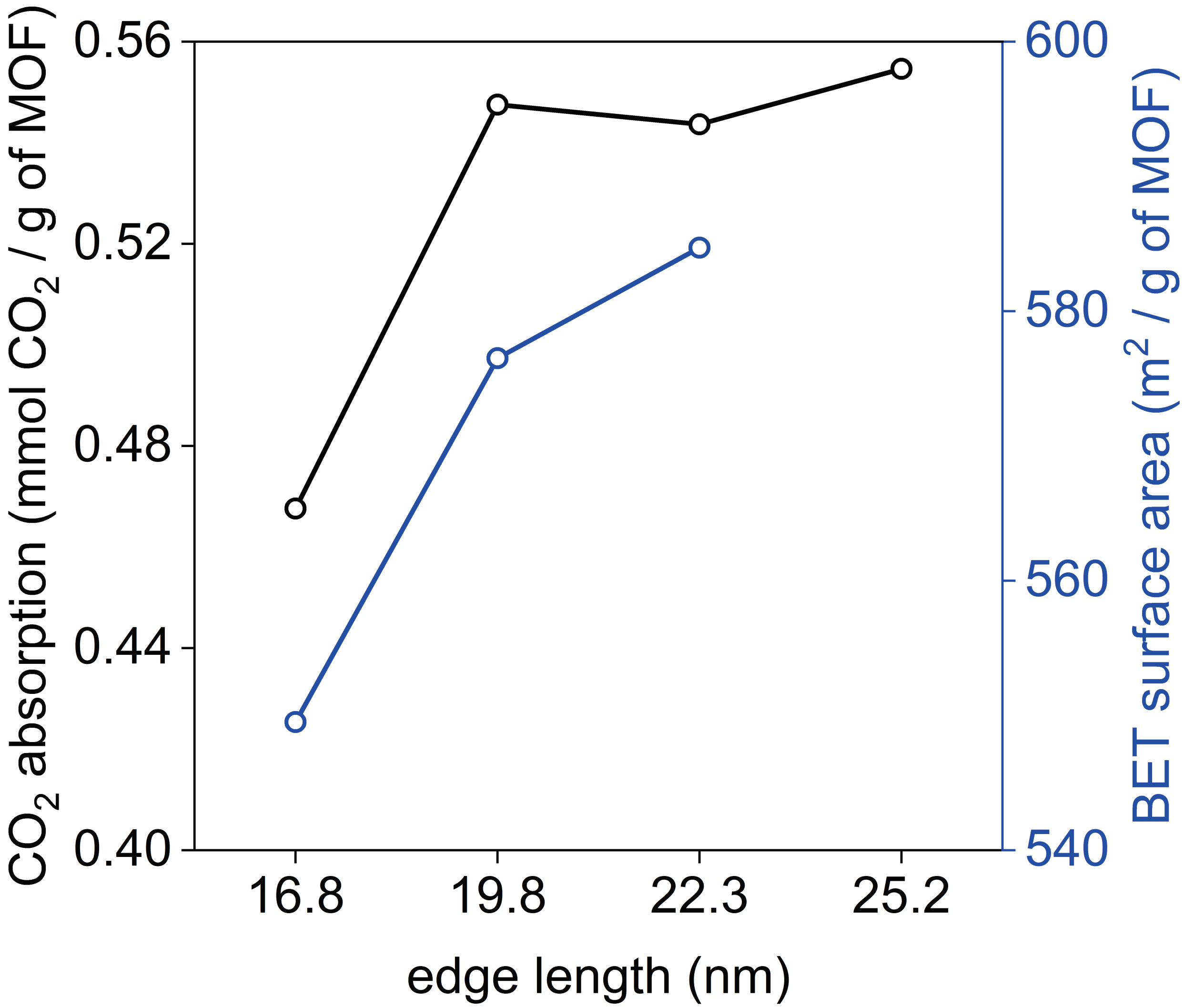

Figure S17. Ligand-corrected gas uptake capacities of ODPA-treated MOFs for CO_2 (black) and for N_2 (blue).

Table S1. Change in BJH adsorption/desorption cumulative pore volume and average pore width upon ODPA treatment.

Edge length (nm)	D (nm)	Pore volume oleate (cm^3/g)	Pore volume ODPA (cm^3/g)	Pore width oleate (nm)	Pore width ODPA (nm)
16.8	22.8	0.513/0.518	0.429/0.425	11.8/9.74	7.12/6.55
19.8	27.0	0.444/0.448	0.355/0.353	11.0/9.15	6.41/5.84
22.3	31.1	0.396/0.387	0.329/0.328	10.6/9.35	6.75/6.22
25.2	35.3	0.255/0.251	nd[a]	12.8/11.1	nd[a]

[a] The yield of the 25.2 nm ODPA capped MOF synthesis was insufficient for this analysis.

SECTION S5: MODELLING

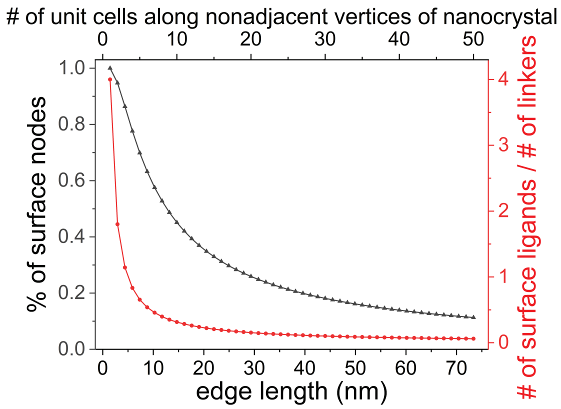

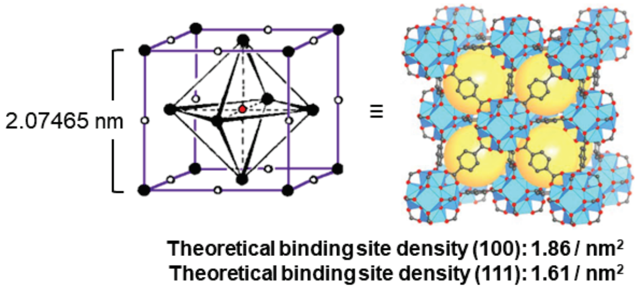

UiO-66 has a cubic unit cell (depicted above). Based on STEM images, the particles appear octahedral. As an octahedral unit cell is not possible, a corrected UiO-66 model is constructed first by building a cube made of cubic unit cells, and then truncating the cube at the vertices, parallel to the [111] facets, a total of 8 times. This results in an octahedron which has a volume that is one sixth of the original volume prior to truncation.

Number of unit cells in cube MOF model	Edge of MOF, prior to truncation (nm)	Length from one vertex to adjacent vertex after truncation (nm)	$\frac{\#\ of\ surface\ ligands}{\#\ of\ linkers}$ prior to truncation (**cube morphology**)	$\frac{\#\ of\ surface\ ligands}{\#\ of\ linkers}$ after truncation (**octahedral morphology**)
1×1×1	2.07	1.47	2.66667	4
2×2×2	4.15	2.93	1.12195	1.8
3×3×3	6.22	4.40	0.71875	1.142857
4×4×4	8.30	5.87	0.52921	0.833333
5×5×5	10.37	7.33	0.41877	0.654545
6×6×6	12.45	8.80	0.34644	0.538462
7×7×7	14.52	10.27	0.29539	0.457143
8×8×8	16.60	11.74	0.25744	0.397059
9×9×9	18.67	13.20	0.22813	0.350877
10×10×10	20.75	14.67	0.2048	0.314286
11×11×11	22.82	16.14	0.1858	0.284585
12×12×12	24.90	17.60	0.17002	0.26
13×13×13	26.97	19.07	0.15671	0.239316
14×14×14	29.05	20.54	0.14533	0.221675

15×15×15	31.12	22.00	0.13549	0.206452
16×16×16	33.19	23.47	0.1269	0.193182
17×17×17	35.27	24.94	0.11933	0.181513
18×18×18	37.34	26.41	0.11262	0.171171
19×19×19	39.42	27.87	0.10662	0.161943
20×20×20	41.49	29.34	0.10122	0.153659
n×n×n	2.07465 n	$\frac{2.07465\text{ n}}{\sqrt{2}}$	$\frac{8n^2+6n+2}{4n^3+2n^2+n-1}$	$\frac{6n^2+12n+6}{2n^3+3n^2+n}$

SECTION S6: SOLUTION PHASE NMR SPECTRA

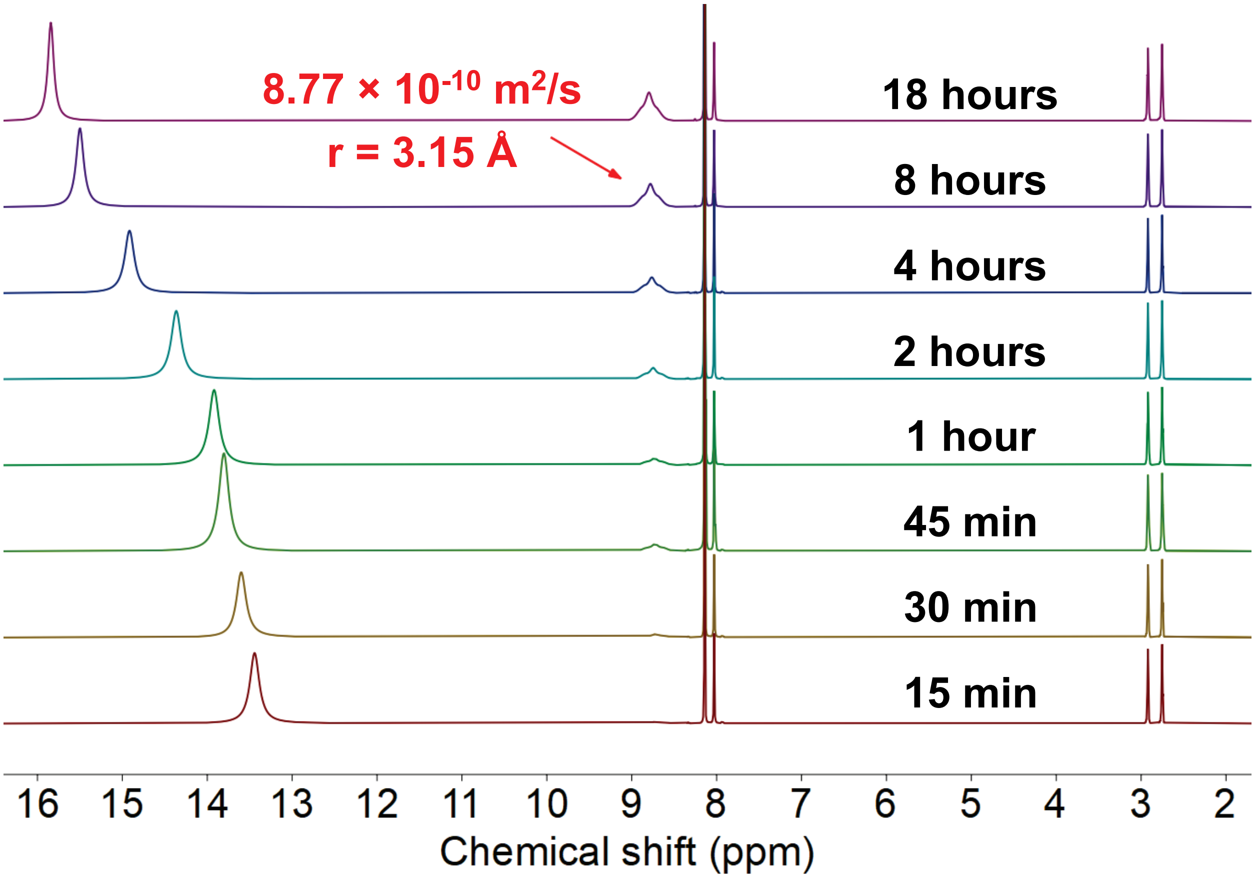

Figure S18. Aliquot analysis of MOF formation in DMF-d_7 using trifluoroacetic acid as modulator. The diffusion coefficients measured for solvent residual peaks are 1.323×10^{-9} and 1.299×10^{-9} m^2/s, ruling out the possibility that the broad signal at ~8.8 ppm originates from MOF nanoparticles.

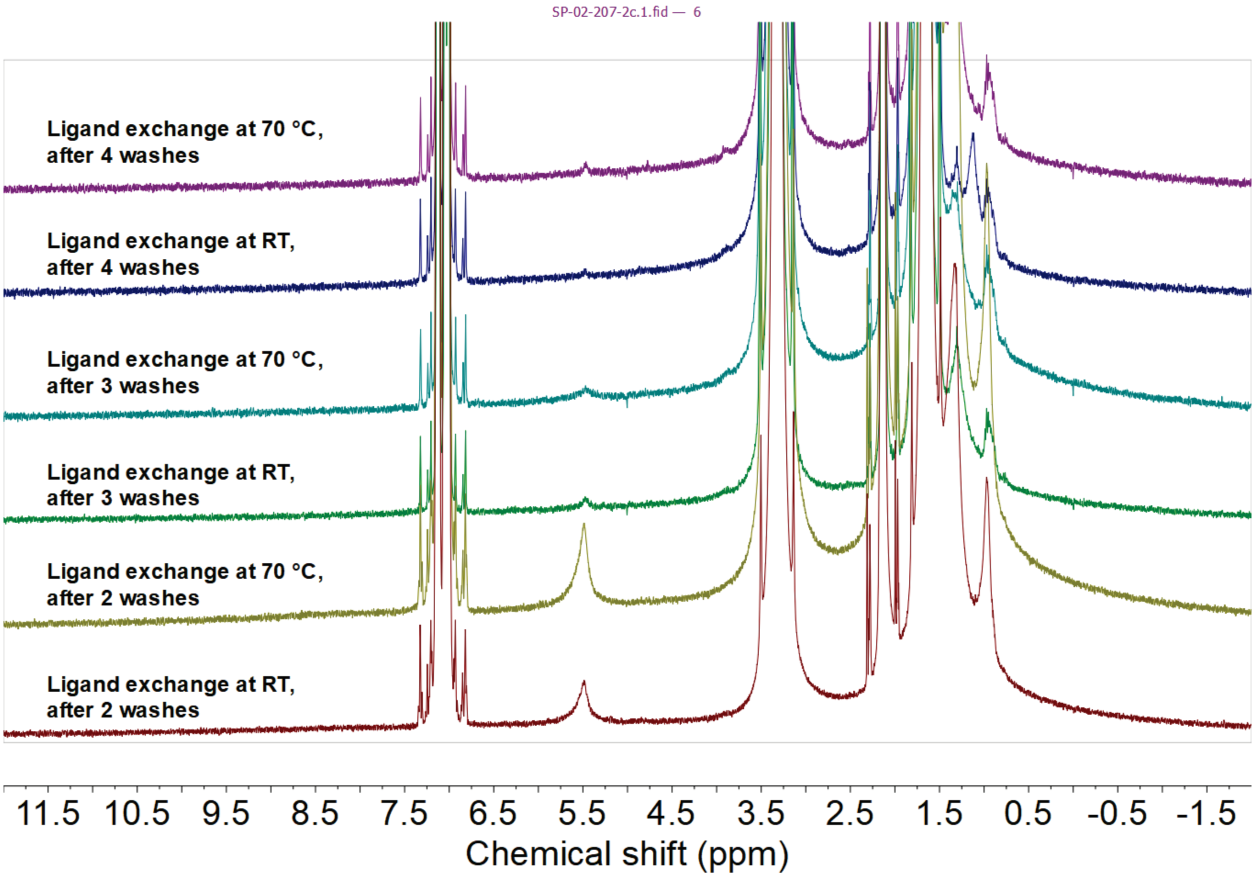

Figure S19. Solution phase ^{1}H NMR spectra of MOF nanocrystals taken in toluene-d_8 after each wash, during the synthesis of oleate capped MOFs.

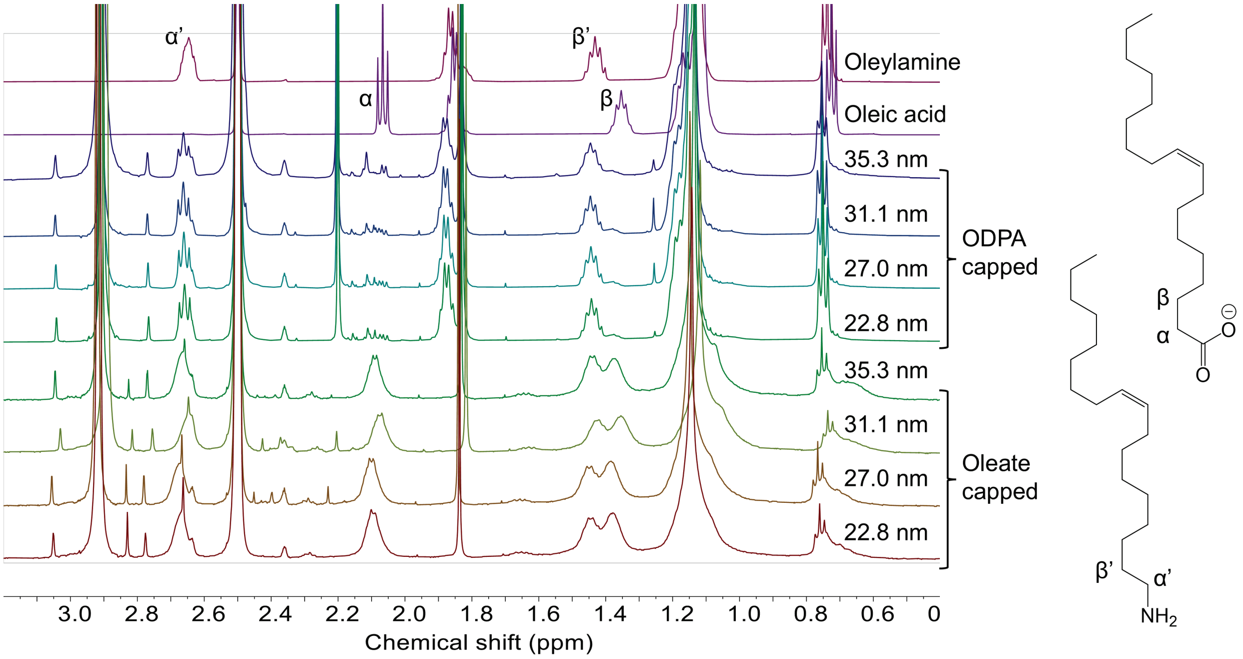

Figure S20. Digestion ^{1}H NMR spectra taken in D_2SO_4/d_6-DMSO. Spectra of pure oleylamine and pure oleic acid taken in D_2SO_4/d_6-DMSO are included at the top for reference.

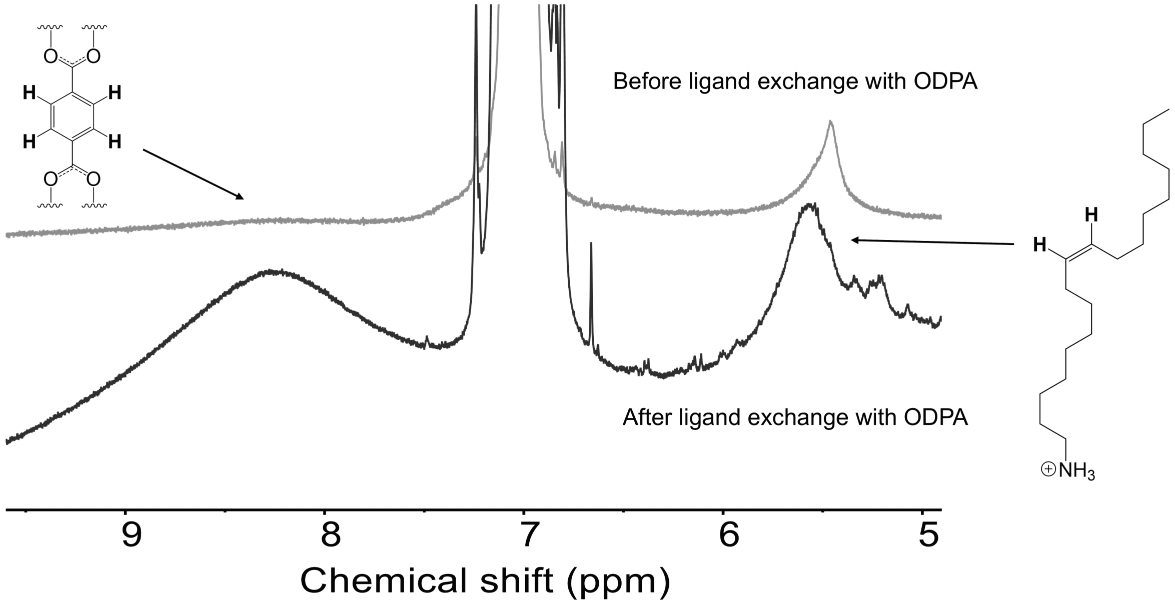

Figure S21. Solution phase ^{1}H NMR spectra of 22.3 nm MOFs taken in toluene-d_8 before (grey trace) and after (black trace) ODPA treatment.

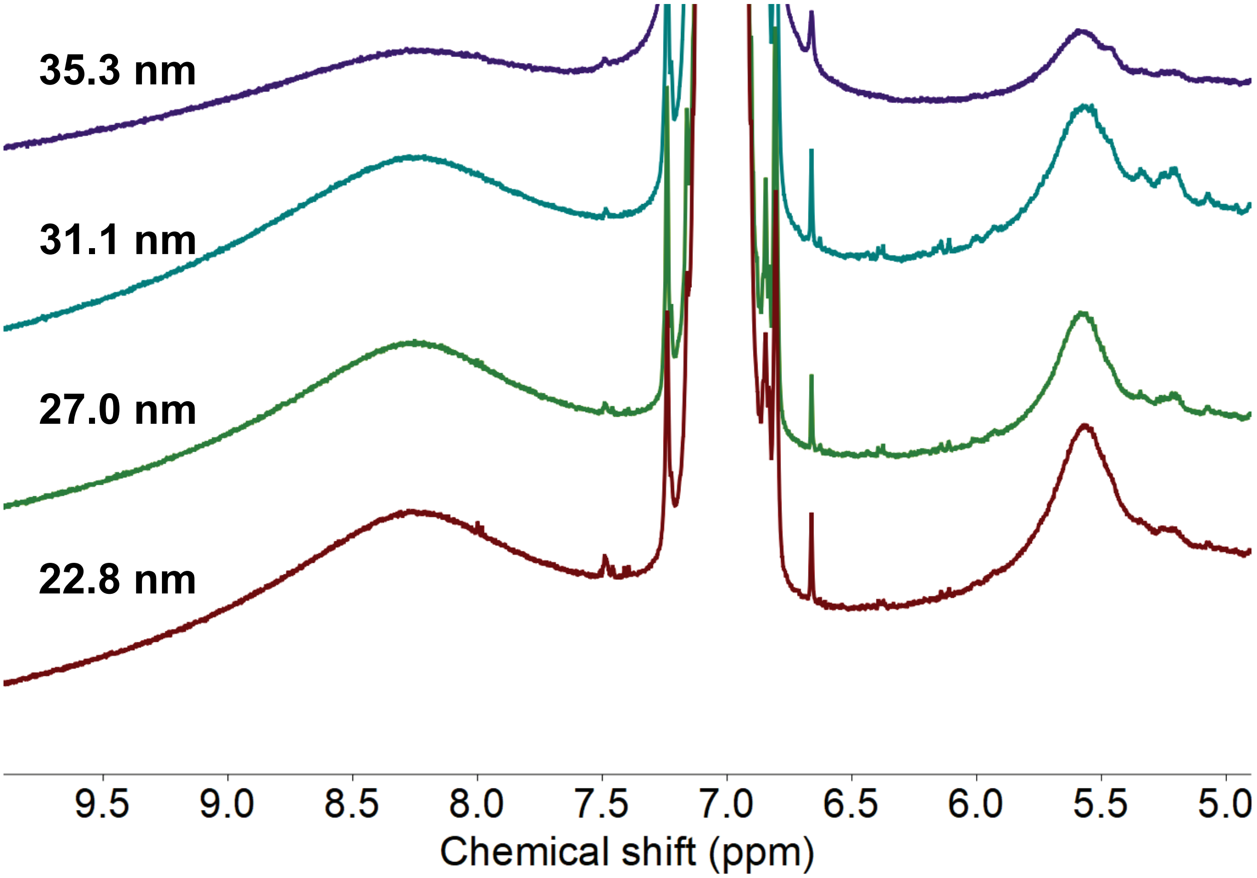

Figure S22. Solution phase ^{1}H NMR spectra of ODPA treated MOFs dispersed in toluene-d_8.

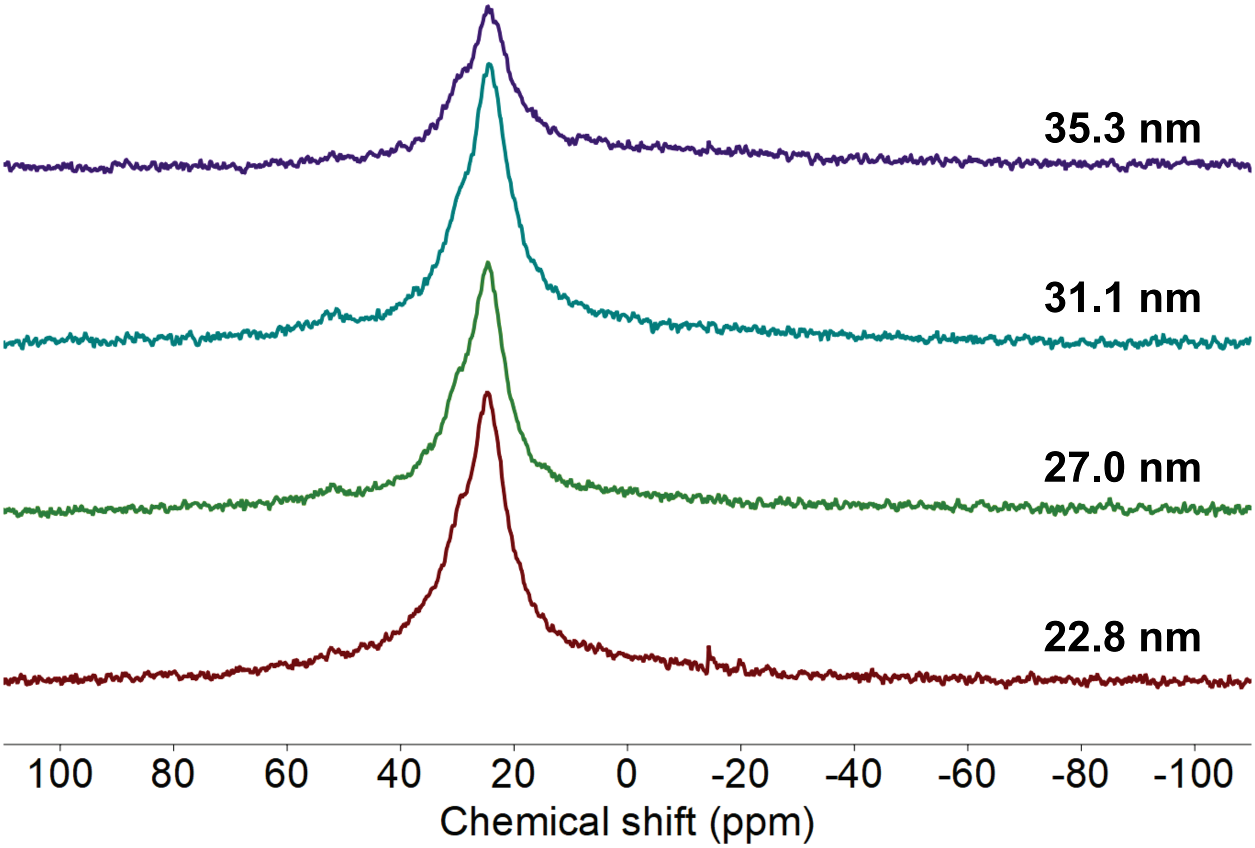

Figure S23. Solution phase ^{31}P NMR spectra of ODPA treated MOFs dispersed in toluene-d_8.

D (nm)	Linker (^1H)	Oleylamine vinylic (^1H)	^{31}P
35.3	899.88 Hz	154.57 Hz	2045.13 Hz
31.1	661.11 Hz	176.97 Hz	1858.04 Hz
27.0	509.30 Hz	136.70 Hz	1573.45 Hz
22.8	655.57 Hz	158.59 Hz	1800.30 Hz

Table S2. Linewidth analysis of solution phase NMR spectra.

SECTION S7: REFERENCES

1. D. Baranov, M. J. Lynch, A. C. Curtis, A. R. Carollo, C. R. Douglass, A. M. Mateo-Tejada and D. M. Jonas, Purification of Oleylamine for Materials Synthesis and Spectroscopic Diagnostics for trans Isomers, *Chem Mater*, 2019, **31**, 1223-1230.
2. G. R. Fulmer, A. J. M. Miller, N. H. Sherden, H. E. Gottlieb, A. Nudelman, B. M. Stoltz, J. E. Bercaw and K. I. Goldberg, NMR Chemical Shifts of Trace Impurities: Common Laboratory Solvents, Organics, and Gases in Deuterated Solvents Relevant to the Organometallic Chemist, *Organometallics*, 2010, **29**, 2176-2179.
3. R. K. Harris, E. D. Becker, S. M. C. De Menezes, R. Goodfellow and P. Granger, NMR nomenclature. Nuclear spin properties and conventions for chemical shifts - (IUPAC recommendations 2001), *Pure Appl Chem*, 2001, **73**, 1795-1818.
4. R. K. Harris, E. D. Becker, S. M. C. De Menezes, P. Granger, R. E. Hoffman and K. W. Zilm, Further conventions for NMR shielding and chemical shifts (IUPAC recommendations 2008), *Pure Appl Chem*, 2008, **80**, 59-84.
5. D. C. Harris, *Quantitative chemical analysis*, W.H. Freeman, New York, 5th edn., 1999.
